# Identifying the Effect of Ursolic Acid Against Triple-Negative Breast Cancer: Coupling Network Pharmacology With Experiments Verification

**DOI:** 10.3389/fphar.2021.685773

**Published:** 2021-11-11

**Authors:** Yubao Zhang, Xiaoran Ma, Huayao Li, Jing Zhuang, Fubin Feng, Lijuan Liu, Cun Liu, Changgang Sun

**Affiliations:** ^1^ School of Basic Medicine, Qingdao University, Qingdao, China; ^2^ First Clinical Medical College, Shandong University of Traditional Chinese Medicine, Jinan, China; ^3^ College of Basic Medical, Shandong University of Traditional Chinese Medicine, Jinan, China; ^4^ Department of Oncology, Weifang Traditional Chinese Hospital, Weifang, China; ^5^ Qingdao Academy of Chinese Medical Sciences, Shandong University of Traditional Chinese Medicine, Qingdao, China

**Keywords:** ursolic acid, triple-negative breast cancer, antiproliferative, network pharmacology, molecular docking

## Abstract

Triple negative breast cancer (TNBC) is a subtype of breast cancer with complex heterogeneity, high invasiveness, and long-term poor prognosis. With the development of molecular pathology and molecular genetics, the gene map of TNBC with distinctive biological characteristics has been outlined more clearly. Natural plant extracts such as paclitaxel, vinblastine, colchicine etc., have occupied an important position in the treatment of hormone-independent breast cancer. Ursolic acid (UA), a triterpenoid acid compound derived from apple, pear, loquat leaves, etc., has been reported to be effective in a variety of cancer treatments, but there are few reports on the treatment of TNBC. This study performed comprehensive bioinformatics analysis and *in vitro* experiments to identify the effect of UA on TNBC treatment and its potential molecular mechanism. Our results showed that UA could not only reduce the proliferation, migration, and invasion in MDA-MB-231 and MDA-MB-468 cell lines with a dose-dependent manner but also induce cell cycle arrest and apoptosis. Meanwhile, we collected the gene expression data GSE45827 and GSE65194 from GEO for comparison between TNBC and normal cell type and obtained 724 DEGs. Subsequently, PLK1 and CCNB1 related to TNBC were screened as the key targets via topological analysis and molecular docking, and gene set enrichment analysis identified the key pathway as the p53 signaling pathway. In addition, quantitative real-time PCR and western blot verified the key genes were PLK1 and CCNB1. *In vivo* and *in vitro* experiments showed that UA could inhibit the growth of TNBC cells, and down-regulate the protein expression levels of PLK1 and CCNB1 by mediating p53 signaling pathway. These findings provide strong evidence for UA intervention in TNBC via multi-target therapy.

## Introduction

TNBC accounts for 10–15% of all breast cancers, and its long-term prognosis is poor compared with that of other breast cancer subtypes (Sharma, 2016). Although incredible progress has been made regarding treatments, including radiotherapy, chemotherapy, surgery, and neoadjuvant therapy, and the survival rate of patients has been gradually increasing, the high recurrence and mortality rates remain serious health threats due to side effects and multidrug resistance (Rubovszky and Horvath, 2017; DeSantis et al., 2019). Therefore, it is imperative to identify and elucidate the molecular mechanisms of novel therapies with fewer side effects.

With the development of molecular pathology and molecular genetics, the gene expression map of TNBC with distinctive biological characteristics is outlined more clearly (Pavelic and Gall-Troselj, 2001; Ingvarsson, 2017). As a subtype of breast cancer with complex heterogeneity, TNBC lacks estrogen receptor, progesterone receptor, and human epidermal growth factor receptor expression in the hormone level, which increases the difficulty of treatment (Nedeljkovic and Damjanovic, 2019). Natural plant extracts such as paclitaxel (Gluz et al., 2018), vinblastine (Casado et al., 2007), colchicine (Lv et al., 2017) etc., have occupied an important position in the treatment of hormone-independent breast cancer (Israel et al., 2018). Ursolic acid (UA) is a natural small-molecule compound derived from the Chinese medicinal herb Hedyotis diffusa (Manayi et al., 2018). It has a wide range of pharmacological activities, including sedative, anti-inflammatory, antibacterial, antidiabetic, anti-ulcer, and blood sugar-lowering effects (Huang et al., 2011; Wang et al., 2017). In recent years, its anti-cancer properties have also been discovered (Huang et al., 2012; Yin et al., 2018; Chan et al., 2019). According to previous reports, UA could inhibit the proliferation of human liver cancer cells by regulating STAT3 activation both *in vivo* and *in vitro* (Liu et al., 2017). It could significantly inhibit the transcription of the angiogenic genes uPA, HIF-1R, VEGF, and IL-8 and reduce ROS production, thereby effectively reducing tumor angiogenesis (Lin et al., 2011). Meanwhile, it has been reported that UA could strengthen the sensitivity of TNBC cells to doxorubicin by interfering with zeb1-as1/mir-186-5p/ABCC1 signaling pathway. (Lu et al., 2021). In addition, UA could also reverse paclitaxel resistance by targeting the miRNA-149-5p/MyD88 axis in MDA-MB-231 cells (Xiang et al., 2019). Although UA is a natural product with an excellent prospect for tumors treatment, there is still a lack of complete understanding of the pharmacological molecular mechanism of UA against TNBC.

Network pharmacology is a drug design approach that encompasses system biology and network analysis to clarify the interaction mechanisms of targets and multi-target drugs from the molecular perspective (Hopkins, 2008). “Multi-target, multi-component, and multi-path” are typical features of natural products, and the network pharmacology approach has been recommended for carrying out more precise studies on the interactions between the active compounds of natural products and diseases (Li et al., 2014). In recent years, comprehensive bioinformatics has been used as a novel tool to study the molecular mechanisms of disease occurrence, and it has also provided great convenience for exploring drug targets (Stratton et al., 2009; Oulas et al., 2019).

In the present study, to clarify the molecular mechanism of UA intervention in TNBC, comprehensive bioinformatics analysis was used to screen for biomarkers related to TNBC, and the validity of the inferences was verified based on molecular docking and *in vitro* experiments.

## Materials and Methods

### Reagents

UA was obtained from the National Institutes for Food and Drug Control (Beijing, China). Cell Counting Kit-8(CCK-8) was obtained from Sigma-Aldrich (Shanghai, China). MDA-MB-231 and MDA-MB-468 cell lines were provided by the Department of Biochemistry and Molecular Biology, Department of Medicine, Qingdao University. Annexin V-FITC Apoptosis Kit was obtained from Becton, Dickinson and Company (New jersey, United States). Goat anti-rabbit horseradish peroxidase (HRP)–conjugated secondary antibodies and Goat anti-mouse horseradish peroxidase (HRP)–conjugated secondary antibodies were obtained from Abcam (United Kingdom). Mouse anti-P53 (catalogue number ab26), rabbit anti-PLK1 (catalogue number ab189139), mouse anti-CCNB1 (catalogue number ab72), and GAPDH (catalogue number ab181602) were purchased from Abcam (United Kingdom).

### Cell Culture

Three cell lines, including Homo sapiens TNBC cell lines MDA-MB-231, MDA-MB-468, and normal cell line HMEC were cultured with the complete medium (10% fetal bovine serum) in an incubator. The culture medium was tested and confirmed to be free of *mycoplasma* contamination.

### Cell Viability Assay

CCK-8 was used to measure the cell viability of MDA-MB-231, MDA-MB-468 cells and HMEC cell treated with UA. At 37°C and 5% CO_2_, 2 × 10^3^ cells were inoculated per well and cultured for 24 h in a 96-well plate. They were placed in a 37°C incubator for 24 h. The cells were exposed to 0 (blank control), 0.1% DMSO (negative control), 15, 20, 25, and 30 μmol/L UA (98% purity) and then incubated for 24, 48, and 72 h, respectively. After treatment, the medium was removed, and 100 μl fresh medium containing 10% CCK-8 was added to each well at 37°C for 4 h. The OD_450_ was measured in a 96-well plate with a Multiskan™ FC Microplate Reader (Thermo Fisher Scientific, China). Three independent experiments were performed.

### Cell Morphology Assay

MDA-MB-231 and MDA-MB-468 cells (5 × 10^5^/well) were added into 6-well plate and cultured overnight in 37°C incubator. Subsequently, the TNBC-cells were treated with UA (0, 15, 20, and 25 μmol/L) for 24 h. The morphological changes of cells were measured and recorded with microscope (100 x).

### 5-Ethynyl-2′-Deoxyuridine Proliferation Assay

MDA-MB-231 and MDA-MB-468 cells (5 × 105/well) were transplanted into 6-well plates and cultured overnight in 37°C incubator and then the cells were treated with UA (0, 15, 20, and 25 μmol/L) for 24 h at 37°C. 1°ml EDU was added to each well and incubated at 37°C for 2 h. PBS containing 4% paraformaldehyde was added to fix the cells for 30°min, and then the cells were treated with glycine (2 mg/ml) and 0.5% triton-100 for 10 min. Cells were treated with 1 ml of 1X Apollo and 1 ml of 1X hoechst33342 at room temperature for 30°min. The average number of three random fields in the sample under fluorescence microscope is used to measure the cell proliferation. Three independent experiments were carried out.

### Wound Healing Assay

MDA-MB-231 and MDA-MB-468 cells (1 × 10^5^ cells/ml) were added to the 6-well plate and incubated overnight. Scrape in a straight line with a 200 μl pipette tip, and then wash the culture wells containing cells 3 times with PBS. The TNBC-cells were treated with different concentrations of UA (0, 15, 20, and 25 μmol/L) for 24 h. The distances that the cells moved were measured and recorded with a microscope (100x).

### Transwell Invasion Assay

Dilute the Matrigel with serum-free medium at a ratio of 1:8 and add it to the upper chamber, place it in a 37°C incubator to coagulate Matrigel, and hydrate it with serum-free medium for 30 min. After adjusting the cell concentration of the suspension to 1 × 10^5^/ml, add 100 μl of cell suspension containing different concentrations of UA (0, 15, 20, and 25 μmol/L) to the upper chamber. A complete medium (10% fetal bovine serum) was added into the lower chamber, and then the TNBC cells were cultured in the incubator for 24 h. Then, aspirate the upper chamber medium and clean the remaining cells in the upper chamber with a cotton swab. After being immersed in paraformaldehyde for 25°min, stained with 1% crystal violet solution for 15°min, and washed 3 times with PBS. Then the microscope was used to observe and count the adherent cells. Three independent experiments were performed.

### Cell Cycle and Apoptosis Assay

MDA-MB-231 and MDA-MB-468 cells were treated with UA (0, 15, 20, and 25 μmol/L) for 24°h, respectively. The cell cycle detection kit (Dojindo, C543) and the Annexin V-FITC/PI dual-labeled flow cytometer kit (BD, 556547) were used to detect the effect of UA on TNBC cell apoptosis and cell cycle by PI staining. The cells were analyzed by flow cytometry using LSRFortessa (BD Biosciences, China).

### Data Source and Processing

Differentially expressed TNBC genes and normal genes were obtained from the GEO database (https://www.ncbi.nlm.nih.gov/geo/, series: gse45827 (Gruosso et al., 2016) and gse65194 (Maire et al., 2013), GLP570). The “affy” package from R studio was used for batch normalization, and mRNA expression data was obtained for 104 samples, including 22 normal tissue samples and 82 TNBC tissue samples. Then, the downloaded gene expression information was normalized, and the gene expression difference between the normal and TNBC samples was identified using the “limma” software package on the R platform.

### Enrichment Analysis and PPI Network

Gene Ontology (GO) enrichment analysis was conducted using the ClusterProfiler software package in the R platform, and *p* < 0.05 was set as the cutoff which indicated biological significance. The DAVID database (Huang et al., 2007) was used to annotate potential targets, and the signal pathway was obtained. A cutoff value *p <* 0.05 was set as the standard for constructing the target interaction network.

A protein-protein interaction (PPI) network for TNBC-related DEGs was constructed. DEGs were entered into STRING, a protein-protein prediction database (Szklarczyk et al., 2015) for analysis, and the STRING database predicted the interactions between the proteins. In the interface, the minimum interaction threshold was set to 0.400, and the species was set as “Homo sapiens”. (Szklarczyk et al., 2017). The target interaction data were entered into the Cytoscape 3.7.1 software, and the topology analysis was performed by CytoNCA plug-in at the PPI network (Shannon et al., 2003). Then, the afore-mentioned modular analysis was used to screen the main nodes with the strongest interactions in the PPI network to identify candidate biomarkers for TNBC.

### Molecular Docking

Hub targets with prognostic value were screened as research objects, and the small-molecule structure of the UA was collected from the PubChem database (Kim et al., 2019) (http://www.pubchem.ncbi.nlm.nih.gov) and saved as mol_2_ format files. The top target-ligand complexes with crystal structures of the hub targeted proteins were collected from the RCSB Protein Data Bank (https://www.rcsb.org/). To further improve the accuracy of the results, SYBYL-X software was used as a virtual docking tool for the batch docking of protein targets and potential active drug ingredients. After the hydrogenation and removal of co-crystallized ligands and water molecules from the protein-ligand compounds, the docking scores were used to assess the affinity of the compound to the candidate targets.

### Prognostic Value Analysis

GEPIA (Tang et al., 2017) (http://gepia.cancer-pku.cn) has 9,736 tumor samples and 8,587 normal samples. The sources for the databases, including TCGA and GTEx, were employed to evaluate the effects of key targets on the overall survival (OS) and recurrence-free survival (RFS) of the patients. *p* < 0.05 was regarded as statistically significant for hub target-related survival.

### Gene Set Enrichment Analysis

We used the Genome Enrichment Analysis (GSEA) version 4.0.0 tool to incorporate candidate biomarkers into the analysis and divide the TNBC chip samples into normal and tumor groups. c2.cp.kegg.v7.2.symbols.gmt was used as the reference gene set, and the degree of random combination was set to 1,000. The detection phenotype was set as a single gene, and the enrichment degrees of the target genes were ranked based on the Pearson coefficient.

### Quantitative Real-Time PCR Analysis

TNBC cells MDA-MB-231 and MDA-MB-468 were treated with UA (0, 15, 20, and 25 μmol/L) for 24 h. The commercial kit (TianGen Biochemical Technology, China) was used to extract total RNA from MDA-MB-231 and MDA-MB-468 cells according to the instructions of manufacturer, and high-capacity cDNA was used to reverse-transcribe 500 ng RNA with a cDNA Reverse Transcription Kit (Thermo Scientific, United States). The expression of PLK1, CCNB1, and P53 genes was measured by real-time PCR using QuantStudio 3 Real-Time PCR Systems (Thermo Fisher Scientific). The primers used were: PLK1 sense, GGC​AAC​CTT​TTC​CTG​AAT​GA; antisense, AATGG- ACCACACATCCACCT; CCNB1 sense, CCA​AAT​CAG​ACA​GAT​GGA​A; antisense, GCC​AAA​GTA​TGT​TGC​TCG​A; and P53 sense, CTG​AGG​TTG​GCT​CTG​ACT​GTA; antisense, GGA​GGA​TTG​TGG​CCT​TCT​TTG.

### Western Blot Analysis

The TNBC cells MDA-MB-231 and MDA-MB-468 were cultured to 70% in the culture dish, treated with UA (0, 15, 20, and 25 μmol/L) for 24 h, and washed with precooled PBS three times using RIPA cell lysis buffer supplemented with PMSF, protease, and phosphatase inhibitors. Total protein was extracted from the treated cells and then subjected to western blot analysis. The western blot was performed according to the manufacturer’s instructions. The same amount of protein (30 µg) was separated by SDS-PAGE, transferred to a PVDF membrane, blotted with 5% BSA at room temperature for 1.5 h, incubated with primary antibody overnight at 4°C, and then combined with HRP-binding to incubate the secondary antibody (1:2000). Finally, the membrane was visualized by ECL Western Blot Substrate.

### Tumor Xenografts Analysis

The specific pathogen free (SPF) BALB/c female nude mice (13–15 g, 6 weeks old) were purchased from Jinan Pengyue Experimental Animal Breeding Co., Ltd. All animal experiments in this project were carried out after being reviewed and approved by the ethics committee of the Animal Experiment Center of Shandong University of Traditional Chinese Medicine (ethics number: SYXK 20170022). The care and use procedures of laboratory animals were also in line with the European Union’s Guide to Laboratory Animals (2010/63/EU). The MDA-MB-231 cells in the logarithmic growth phase were adjusted to a concentration of 1 × 10^7^ cells/ml with phosphate buffered saline (PBS), mixed and injected into the right armpit of BALB/c mice. Each mouse was injected 0.2 ml. The mice were randomly divided into the control group (normal saline) and the UA treatment group (20 mg/kg, 50 mg/kg, and 100 mg/kg), with five mice in each group. Drug injection was performed 1 week later. The tumor volume was measured every 4 days. Mice were euthanized on the day of final treatment, the tumors were removed, weighed and immediately frozen in liquid nitrogen for subsequent Western blot.

### Statistical Analysis

GraphPad Prism eight was used to perform statistical analysis on the data. When appropriate, Student’s t-test or one-way analysis of variance (ANOVA) was performed for statistical analysis, and the data are expressed as the mean ± standard deviation (SD). For all statistical analyses, *p < 0.05, **p < 0.01, and ***p < 0.001 were considered significant differences.

## Results

### Effect of UA on Viability of Typical Cells

**FIGURE 1:**
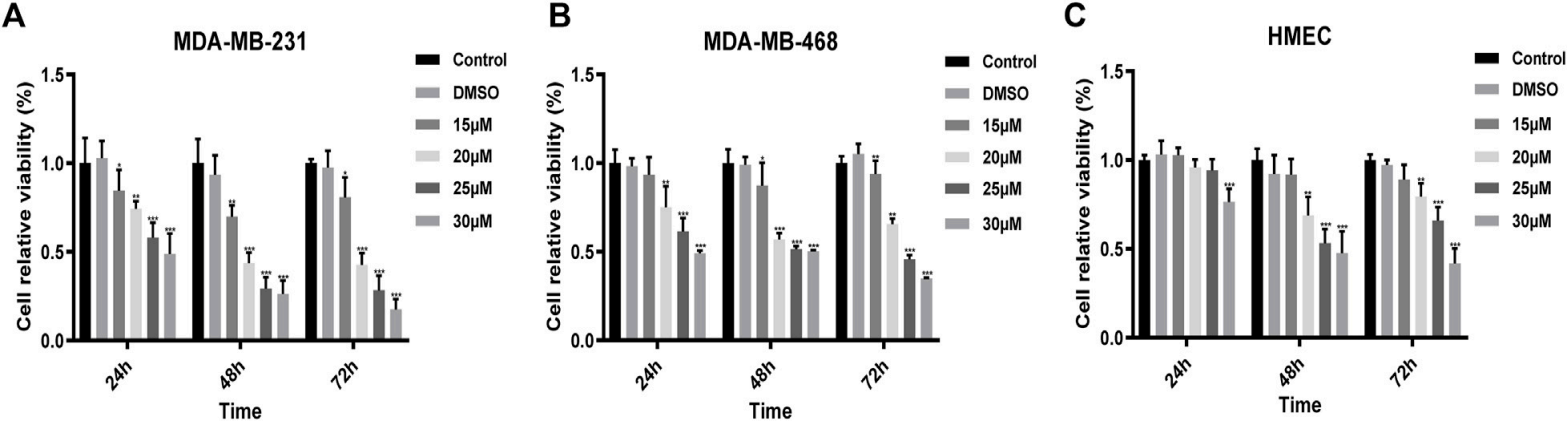
The viabilities of **(A)** MDA-MB-231; **(B)** MDA-MB-468; **(C)** HMEC cells treated with UA (0, 15, 20, and 25 μmol/L) determined by CCK8 assays. Negative control: cells treated with 0.1% DMSO. Data are presented as the mean ± standard deviation (*n* = 3), *p < 0.05, **p < 0.01, and ***p < 0.001 compared with negative control.

To evaluate the inhibitory effect of UA on cells, we selected the MDA-MB-231, MDA-MB-468, and HMEC cell lines, and these cell lines were treated with UA (0, 15, 20, 25, and 30 μmol/L) for 24, 48, and 72 h, separately, then the cell viabilities were detected using CCK8 assays. The results showed that compared with the negative control, UA has a significant inhibitory effect on MDA-MB-231 and MDA-MB-468 cells, and it was time and dose-dependent (Figures 1A,B). However, UA showed lower effect on human breast epithelial HMEC cells (Figure 1C).

### Effect of UA on the Morphology and Proliferation of TNBC Cells

**FIGURE 2:**
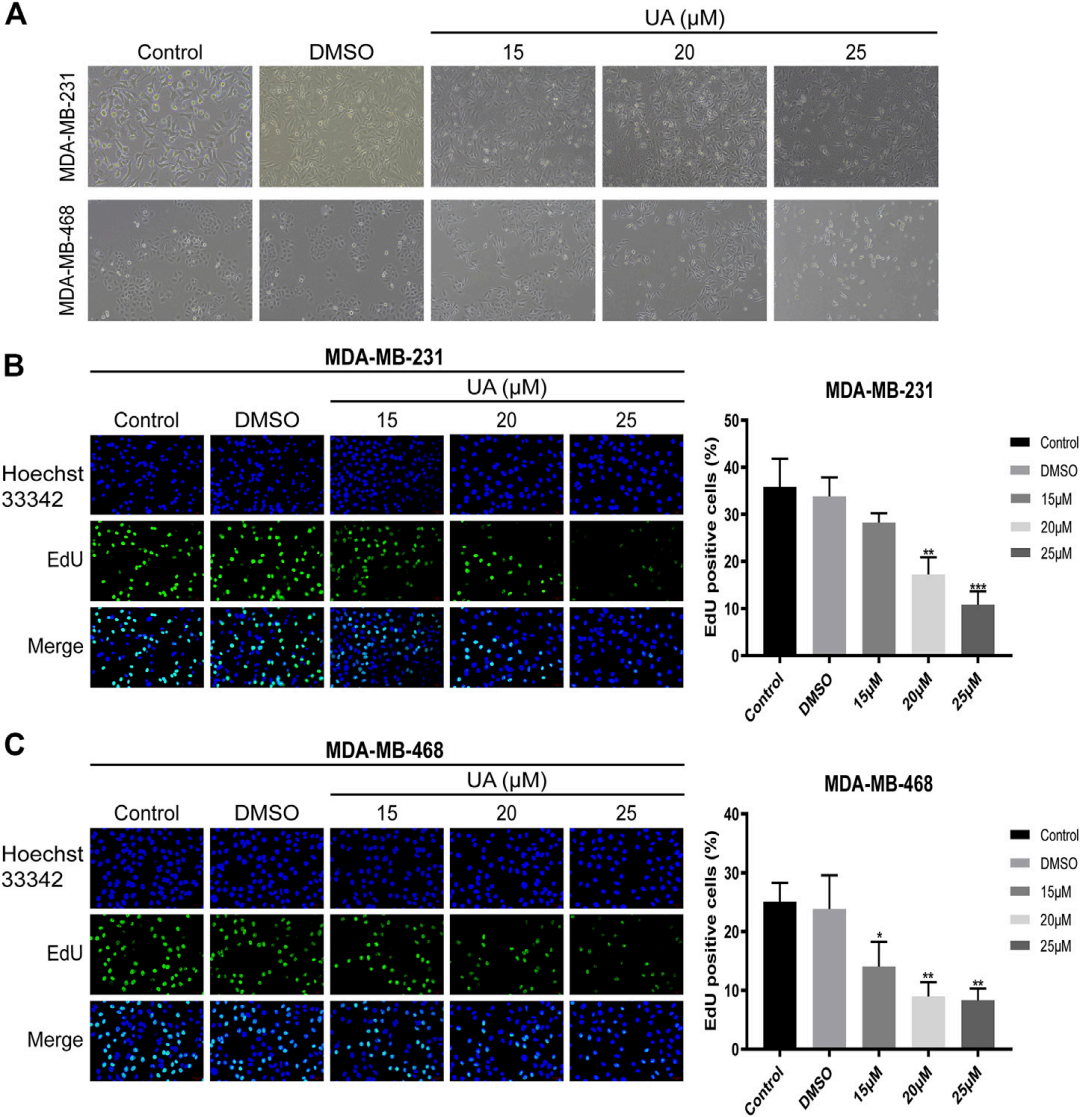
Effect of UA on the morphology and proliferation of MDA-MB-231 and MDA-MB-468 cells. **(A)** Cell morphology of MDA-MB-231 and MDA-MB-468 cells after UA treatment for 24 h. The Phase-contrast images were observed with inverted microscope by a magnification ×100. **(B,C)** Edu staining assay was performed to detect the DNA replication ability of MDA-MB-231 and MDA-MB-468 cells after UA (0, 15, 20, and 25 μmol/L) treatment for 24 h. Data are presented as the mean ± standard deviation (*n* = 3), *p < 0.05, **p < 0.01, and ***p < 0.001 compared with control.

The morphological changes of MDA-MB-231 and MDA-MB-468 cells treated with UA (0, 15, 20, and 25 μmol/L) for 24 h were observed under the microscope. The results showed that compared with the negative control, the number of cells in UA treatment group decreased significantly, with the 25 μmol/L treatment group show a more significant decrease. In addition, the characteristic morphology of the cells gradually disappeared, and some cells began to shrink (Figure 2A). To further explore the effect of UA on the proliferation of TNBC cells, EDU staining and Hoechst33342 staining were performed. After 24 h of UA treatment, the numbers of actively proliferating cells in MDA-MB-231 and MDA-MB-468 cells were significantly lower than that the negative control group, and the numbers of green fluorescent cells labeled with EDU were significantly reduced after UA treatment (Figures 2B,C).

### Effect of UA on the Migration and Invasion of TNBC Cells

**FIGURE 3:**
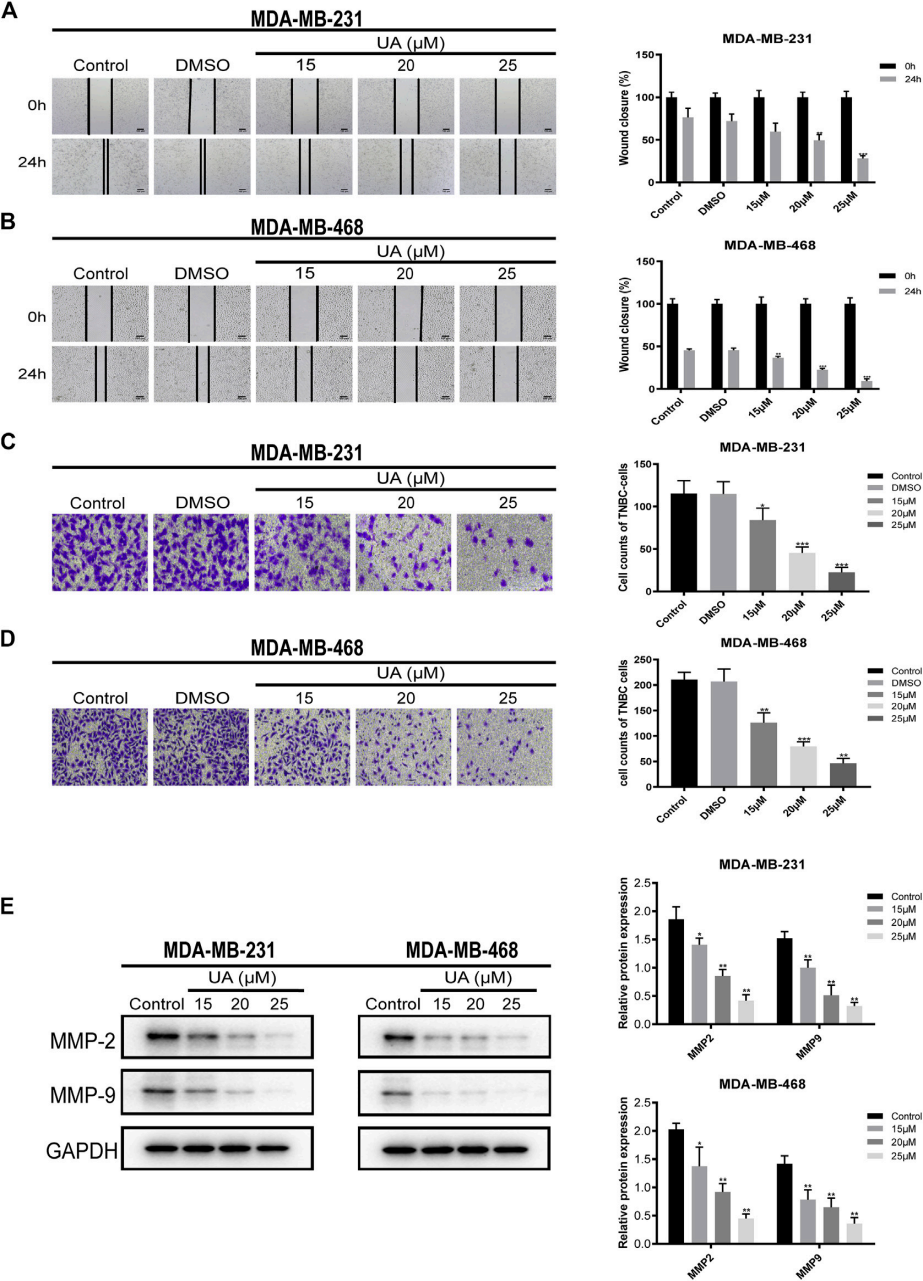
UA inhibits the migration and invasion of MDA-MB-231 and MDA-MB-468 cells. **(A,B)** Wound healing assay was performed to detect the migration abilities of MDA-MB-231 and MDA-MB-468 cells after UA (0, 15, 20, and 25 μmol/L) for 24 h. **(C,D)** The invasion abilities of MDA-MB-231 and MDA-MB-468 cells were measured by Transwell assay. **(E)** The expression levels of MMP2 and MMP9 were monitored using western blot analysis. Data are presented as the mean ± standard deviation (*n* = 3), *p < 0.05, **p < 0.01, and ***p < 0.001 compared with control.

To further study the migration and invasion abilities of MDA-MB-231 and MDA-MB-468 cells treated with UA (0, 15, 20, and 25 μmol/L), wound healing and invasion experiments were performed. In addition, the expressions of MMP2 and MMP9 in TNBC cells has also been further detected, which were considered to have a high correlation in tumor migration and invasion events. The results indicated that UA inhibited the migration ability of MDA-MB-231 and MDA-MB-468 cells in a concentration-dependent manner (Figures 3A,B). Compared with the negative control, MDA-MB-231 and MDA-MB-468 treated with UA (0, 15, 20, and 25 μmol/L) exhibited significantly reduced infiltration of the lower chamber, indicating that UA was pharmacologically active in inhibiting the migration and invasion abilities of MDA-MB-231 and MDA-MB-468 cells (Figures 3C,D). As shown in Figure 3E , compared with the control groups, the protein expressions of MMP2 and MMP9 were significantly reduced in UA-treated (15, 20, and 25 μmol/L) groups. The above results suggested that UA might inhibit the migration and invasion of TNBC cells by mediating MMP2 and MMP9.

### Effect of UA on the Cell Cycle and Apoptosis of TNBC Cells

**FIGURE 4:**
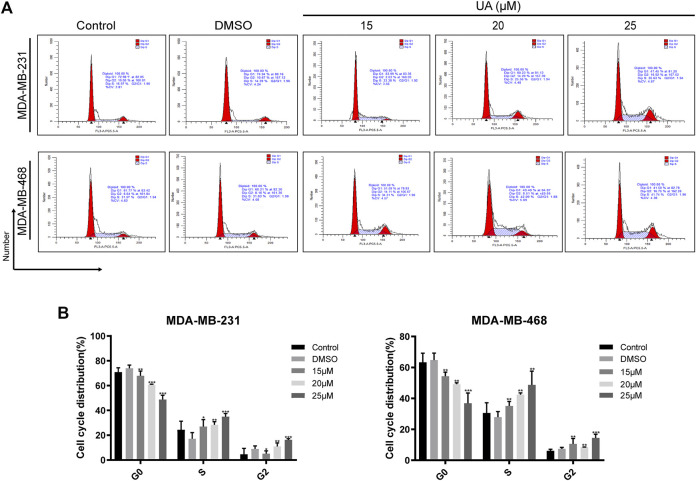
UA induces cell cycle arrest in MDA-MB-231 and MDA-MB-468 cells. **(A)** Flow cytometry was performed to detect the cell cycle distribution of MDA-MB-231 and MDA-MB-468 cells treated with UA (0, 15, 20, and 25 μmol/L) for 24 h. **(B)** Percentage of MDA-MB-231 and MDA-MB-468 cells in each phase. Data are presented as the mean ± standard deviation (*n* = 3), *p < 0.05, **p < 0.01, and ***p < 0.001 compared with control.

**FIGURE 5:**
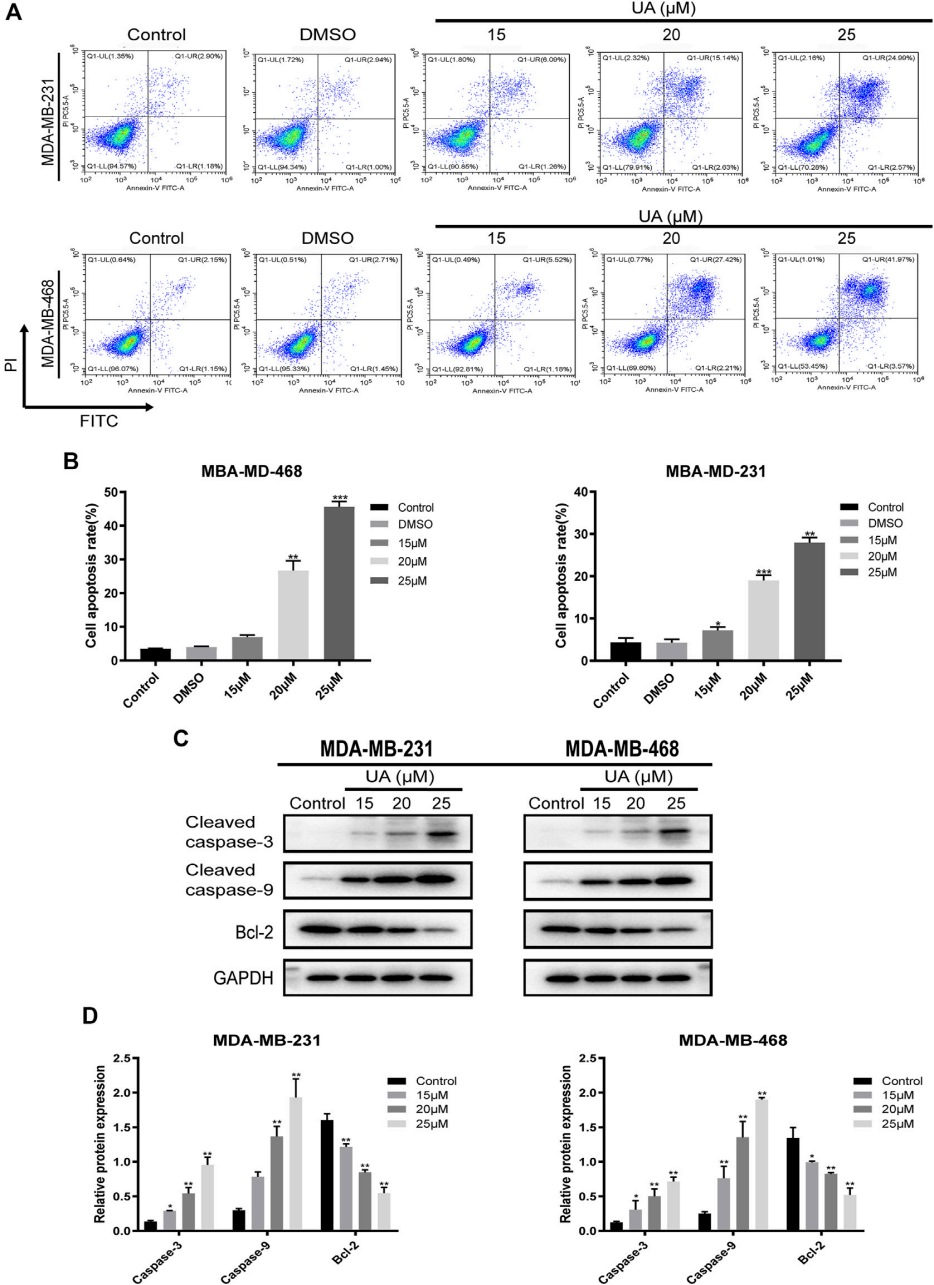
UA induces apoptosis in MDA-MB-231 and MDA-MB-468 cells. MDA-MB-231 and MDA-MB-468 cells were treated with UA (0, 15, 20, and 25 μmol/L) for 24 h. **(A)** Apoptosis assays of TNBC cells were detected via flow cytometry in annexin V-FITC/PI stained, and quantifications of apoptosis in MDA-MB-231 and MDA-MB-468 were shown **(B)**. **(C)** The expression levels of cleaved caspase-3, cleaved caspase-9 and Bcl-2 were monitored using western blot analysis. **(D)** Quantitation of the expressions of cleaved caspase-3, cleaved caspase-9 and Bcl-2. Data are presented as the mean ± standard deviation (*n* = 3), *p < 0.05, **p < 0.01, and ***p < 0.001 compared with control.

The effect of UA on the cell cycle and apoptosis was detected using flow cytometry. Compared with the negative control group, the ratio of cells in G1/G0 phase of MDA-MB-231 and MDA-MB-468 cells treated with UA (0, 15, 20, and 25 μmol/L) was down-regulated, while the ratio of cells in G2/M phase were significantly increased (Figure 4). After 24 h of UA (0, 15, 20, and 25 μmol/L) treatment, the percentage of MDA-MB-231 cells in G2/M phase was 4.647 ± 4.698, 5.130 ± 2.213, 10.980 ± 2.797, 16.280 ± 0.895, respectively, and the percentage of MDA-MB-468 cells in G2/M phase was 6.110 ± 0.970, 10.590 ± 3.495, 8.403 ± 0.150, 14.440 ± 2.372, respectively. In the apoptosis assay, the MDA-MB-231 and MDA-MB-468 cells were stained with Annexin V-FITC and PI after 24 h of treatment with UA (0, 15, 20, and 25 μmol/L), and the degree of apoptosis was measured. As shown in Figure 5, the percentage of apoptosis of TNBC cells was significantly increased. These results suggest that UA could induces S phase arrest and apoptosis of MDA-MB-231 and MDA-MB-468 cells in a dose-dependent manner. To further analyze the apoptosis of TNBC cells treated with UA, we measured the expression of apoptosis-related signature proteins. Compared with the control groups, the protein expressions of cleaved caspase3 and cleaved caspase9 increased significantly after UA (15, 20, and 25 μmol/L) treatment. Meanwhile, Bcl-2 was remarkably reduced in UA-treated groups compared with the control groups.

### Identification of Differentially Expressed Genes

**FIGURE 6:**
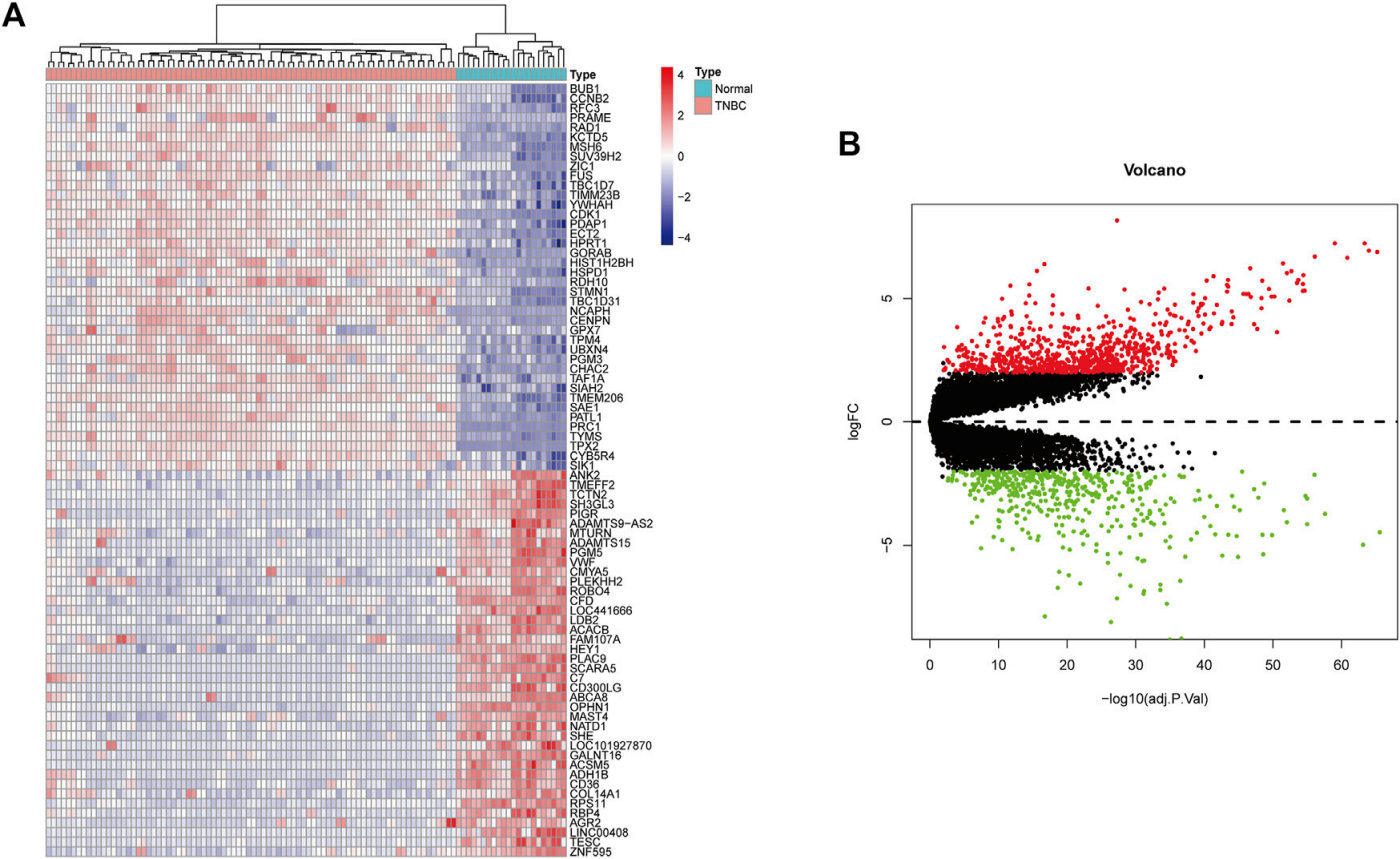
**(A)** Heatmaps show the expression of differential genes in TNBC samples. Red indicates high gene expression and blue indicates low gene expression. **(B)** Volcano map of differentially expressed genes in BC. The red dots represent significantly up-regulated genes, the green dots represent significantly down-regulated genes.

In this study, gse45827 and gse65194 datasets were downloaded from the GEO database. Then, |log2-fold change| ≥2.5 and *p* < 0.01 were used as the screening filter conditions, the differential expression between 22 normal tissues and 82 TNBC tissues was evaluated, and 724 DEGs were identified using the R package. The top 40 up-regulated and down-regulated genes with the most significant differences were selected separately for subsequent analysis (Figure 6).

### Enrichment Analysis and PPI Network Construction

**FIGURE 7:**
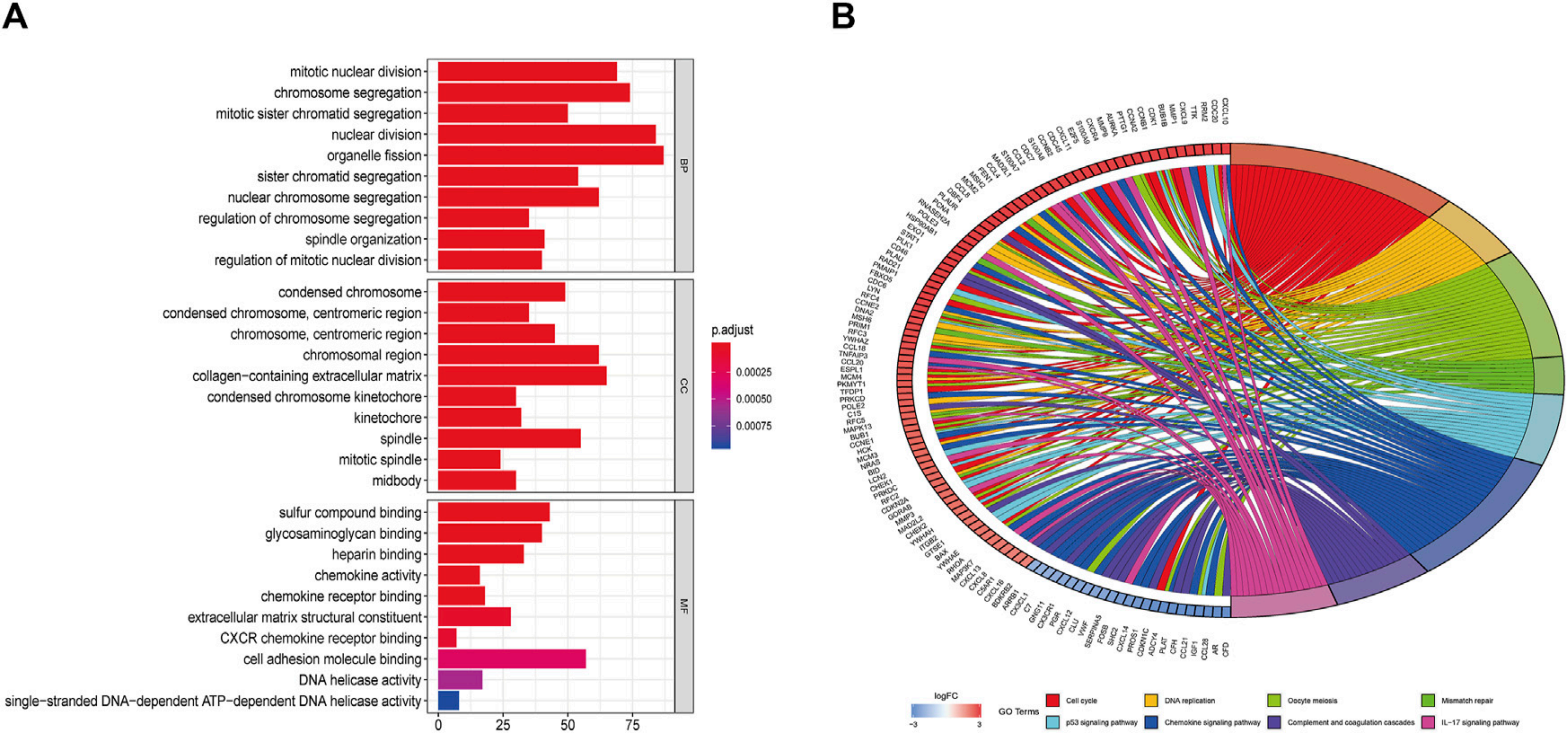
**(A)** GO enrichment (BP, MF and CC) for DEGs (*p* < 0.05). **(B)** Circle plot of related KEGG terms and DEGs belonging to relevant signal pathway (*p* < 0.05).

To further study the differentially expressed genes in TNBC, the R package and DAVID-database were used to conduct GO and KEGG enrichment analyses on differentially expressed genes. *p* < 0.05 was used as the cut-off criterion, and GO analysis was performed on the above-mentioned differentially expressed genes to clarify their molecular function, biological process, and cell composition (Figure 7A). The enrichment analysis showed that the biological processes mainly included mitotic nuclear division, chromosome segregation, organelle fission, and other processes related to cell mitosis. The molecular functions were related to cell activity, such as cell adhesion molecule binding, glycosaminoglycan binding, heparin binding, and chemokine receptor binding. The cell components were related to the chromosomal region, collagen-containing extracellular matrix, condensed chromosome, and centromeric region. In the KEGG pathway enrichment analysis, *p* < 0.01 was set as the filter condition; the analysis showed that the BP-related DEGs were mostly involved in the cell cycle, DNA replication, mismatch repair, and the P53 signaling pathway (Figure 7B).

**FIGURE 8:**
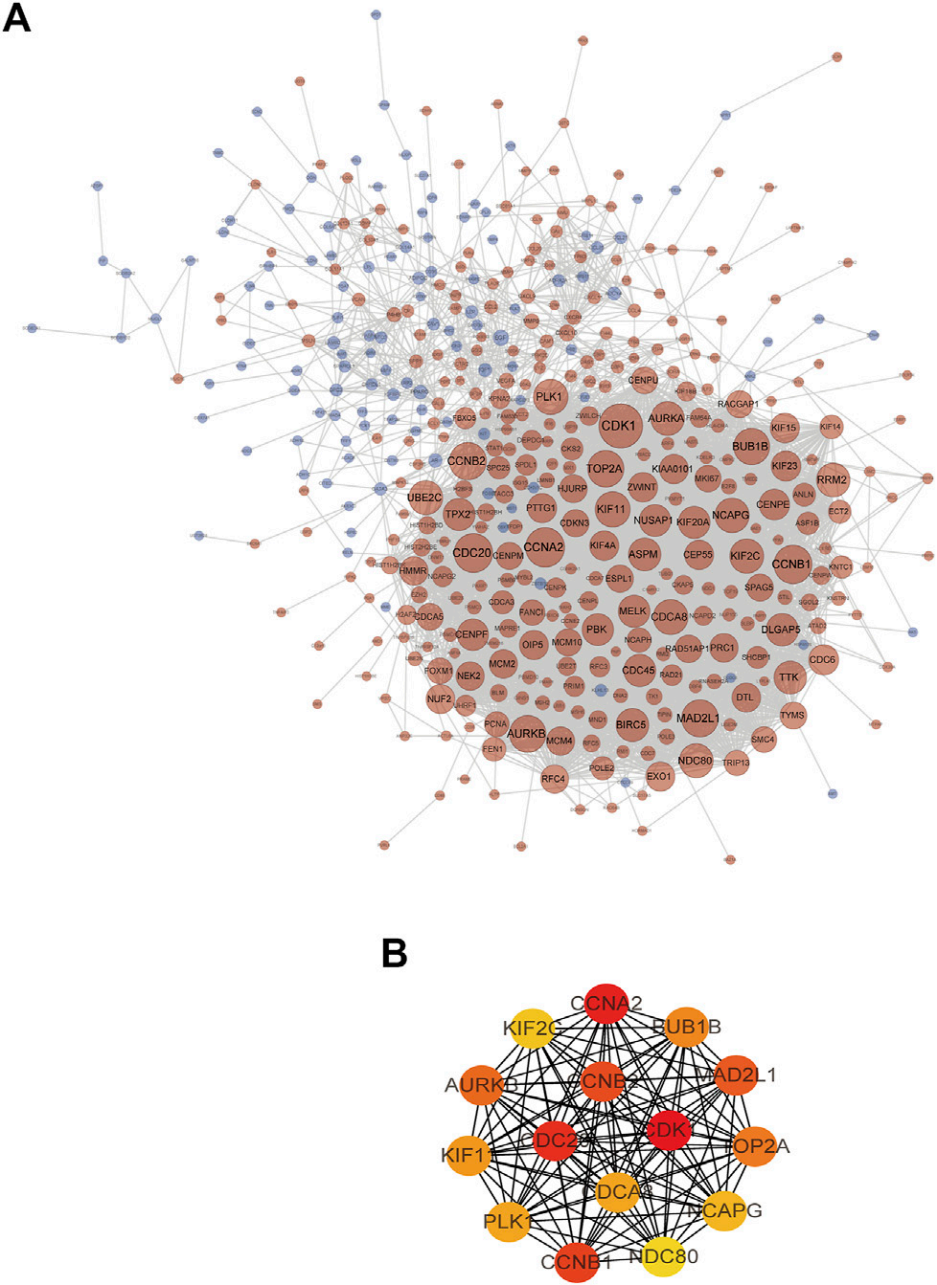
**(A)** The TNBC target network contains 445 nodes and 5,011 edges (blue nodes represent low-expression targets, red nodes represent high-expression targets, and the size of nodes increases with the range of connectivity). **(B)** PPI interaction network of candidate targets (red indicates targets with high connectivity and green indicates targets with low connectivity).

The construction of the PPI network is a rapid method to analyze the interactions between DEGs. String and Cytoscape software were used to build a protein-protein interaction network contain 445 nodes and 5,011 edges (Figure 8A). The plug-in CytoNCA was used for topological analysis of the PPI network. Based on the topological analysis results of the comprehensive ranking by screening degrees and nodes with the highest value were identified, and 15 DEGs were determined as the targets for further analysis (Figure 8B), including CDK1 (cyclin-dependent kinase 1), CCNA2 (cyclin A2), CDC20 (cell division cycle 20), CCNB1 (cyclin B1), CCNB2 (cyclin B2), MAD2L1 (mitotic arrest deficient-like 1), AURKB (aurora kinase b), TOP2A (topoisomerase II alpha), BUB1B (budding uninhibited by benzimidazoles 1 homolog beta), KIF11 (kinesin family member 11), PLK1 (polo-like kinase 1), CDCA8 (cell division cycle associated 8), NCAPG (non-SMC condensin I complex, subunit G), KIF2C (kinesin family member 2c), and UBE2C (ubiquitin-conjugating enzyme e2c).

### Identification of Module by Molecular Docking Analysis

**FIGURE 9:**
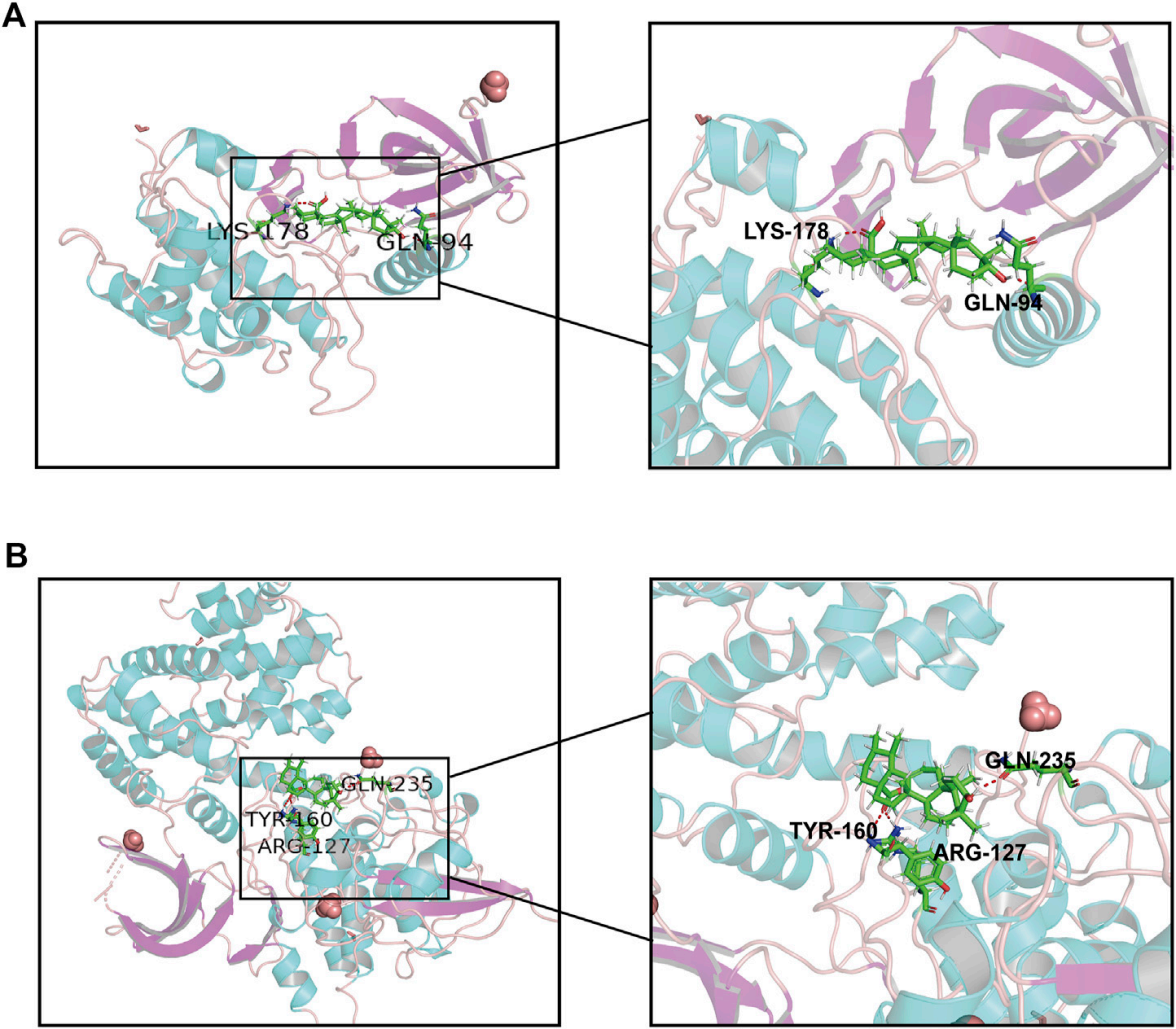
Molecular docking of UA and hub proteins. **(A)** PLK1, **(B)** CCNB1. The green structure represents the small molecule compound, and the red structure represents the binding site of the compound to the hub proteins.

Molecular docking as an irreplaceable computer simulation method in the field of small molecular compounds research and development for studying the binding mode of ligands and receptors and the mechanism of interaction between molecules (Alonso et al., 2006). to further explore the interaction between UA and 15 targets, a molecular docking model of UA and DEGs was constructed, and a docking score greater than five was considered meaningful (Supplementary Table S1). The results showed that PLK1 (id:2RKU, score: 5.9357) and CCNB1 (id: 6GU3, score: 5.2448) had the highest scores in the UA compound model, and the interaction between the residues of the UA small-molecule and hydrogen bond was stable. (Figure 9).

### Identification of Prognostic Value Analysis

**FIGURE 10:**
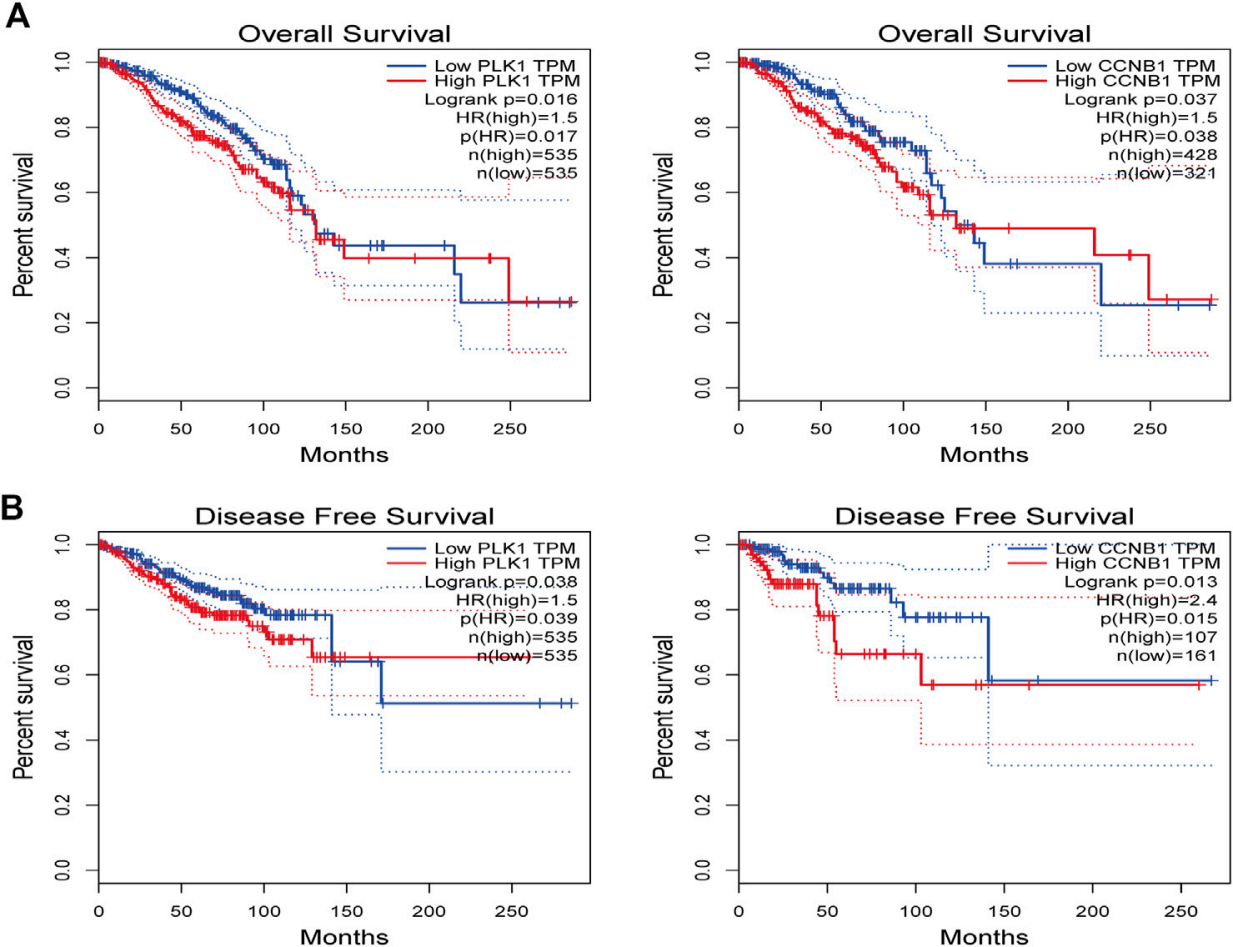
The GEPIA platform was performed to explore the prognostic value of hub targets from TNBC. **(A)** Prognostic analysis for PLK1, **(B)** Prognostic analysis for CCNB1. Log-rank *p* < 0.05 was considered statistically significant.

The bioinformatics analysis tool in the GEPIA platform was used to screen the relationship between key targets and overall survival (OS) as well as key targets and recurrence-free survival (RFS) in breast cancer patient samples (Figure 10). The results showed that high expression of CCNB1 was negatively correlated with poor patient prognosis (*p* < 0.05).

### Identification of Gene Set Enrichment Analysis

**FIGURE 11:**
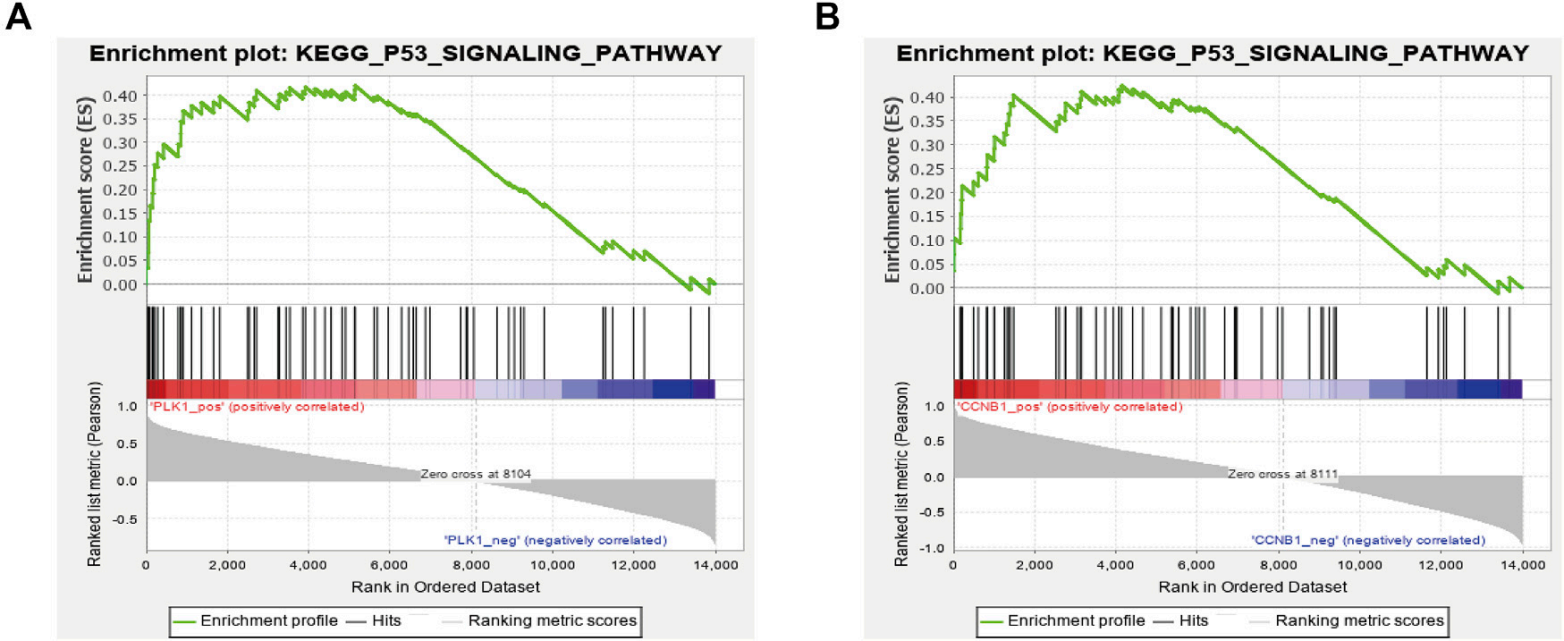
GSEA enrichment analysis with a single gene as the phenotype shows that the expression of PLK1 **(A)** and CCNB1 **(B)** was related to the P53 signaling pathway.

A single gene set enrichment analysis (GSEA) was performed to explore the signaling pathways enriched by these hub genes. The GSEA analysis showed that PLK1 and CCNB1 were both accumulated significantly in the p53 signaling pathway, which was consistent with the previous KEGG enrichment results (Figure 11). Therefore, PLK1, CCNB1, and p53 were used as the core targets for subsequent experimental verification.

### Validation of Key Genes by qRT-PCR and Western Blot

**FIGURE 12:**
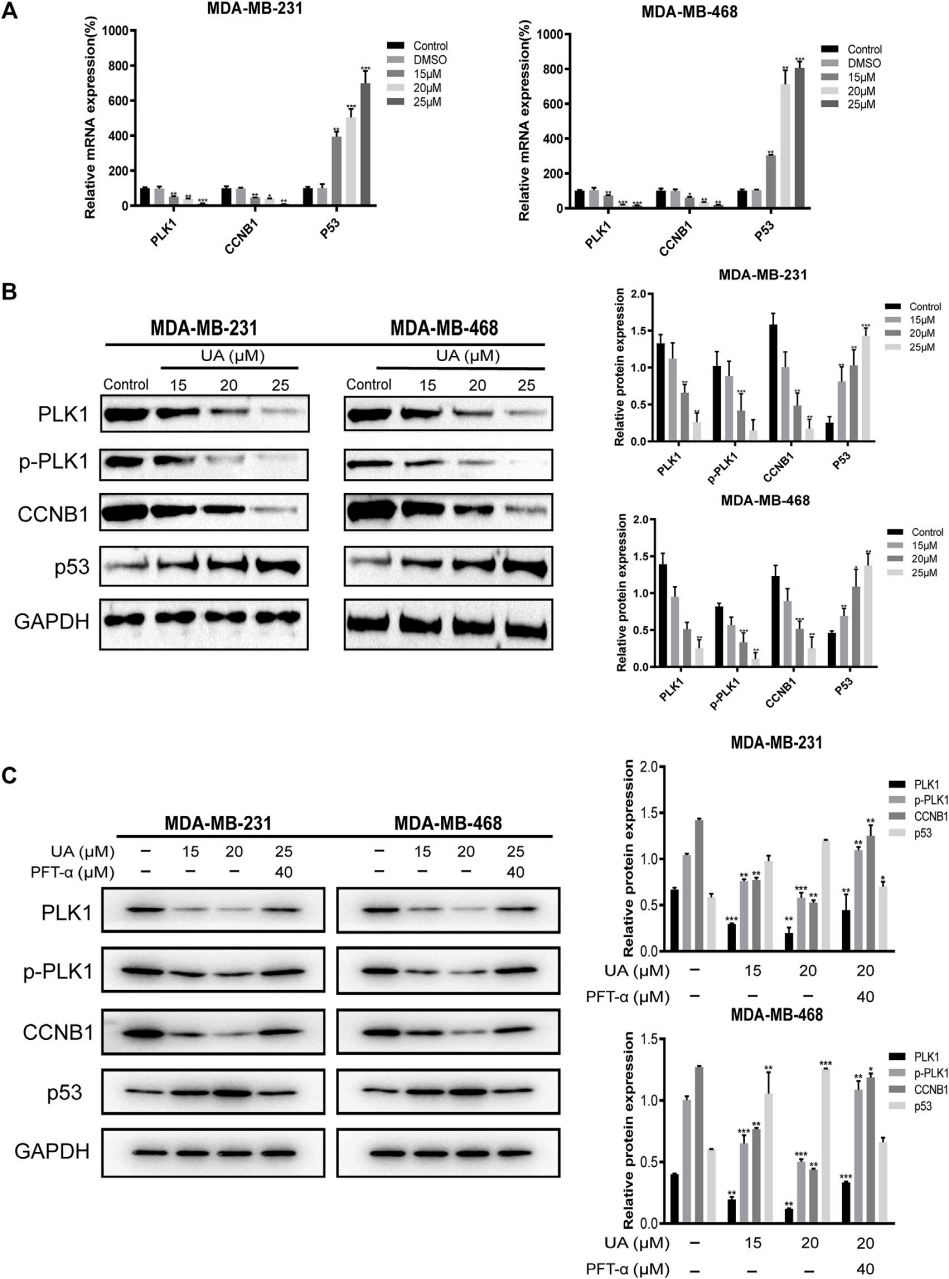
Quantitative Real-Time PCR and Western blot analysis for the expressions of hub targets treated with UA (0, 15, 20, and 25 μmol/L) for 24 h. **(A)** Quantitative Real-Time PCR analyses were performed for *PLK1*, *CCNB1*, and *P53* mRNA expressions in MDA-MB-231 and MDA-MB-468 cells. **(B)** Western blot analyses were performed for PLK1, p-PLK1, CCNB1, and P53 protein expressions in MDA-MB-231 and MDA-MB-468 cells. **(C)** Western blot analysis of PLK1, p-PLK1, CCNB1, and p53 protein expression after adding PFT-α. Data are presented as the mean ± standard deviation (*n* = 3), *p < 0.05, **p < 0.01, and ***p < 0.001 compared with control.

To determine the expression of critical biomarkers in TNBC cells after drug treatment, qRT-PCR and western blot analysis were performed. The results showed that after UA (0, 15, 20, and 25 μmol/L) treatment, the mRNA expression of *PLK1*, *CCNB1*, and *p53* in MDA-MB-231 and MDA-MB-468 cells were concentration dependent. In both cell lines, *PLK1* and *CCNB1* decreased with increasing concentration, while the expression of *p53* increased significantly with increasing concentration (*p* < 0.01) (Figure 12A). Then, the effect of UA on PLK1, p-PLK1, CCNB1, and p53 was determined at the level of protein expression by western blot analysis. In UA-treated (15, 20, and 25 μmol/L) groups, the protein expression of PLK1, p-PLK1, and CCNB1 was significantly reduced compared with the control groups. Besides, the protein expression of p53 also increased significantly with increasing dose, which was consistent with the qRT-PCR results (Figure 12B). To further explore the critical function of p53 in UA affecting TNBC cells, we used Pifithrin-α (PFT-α) as a p53 inhibitor to verify the hypothesis. As shown in Figure 12C , the expression levels of PLK1, p-PLK1, and CCNB1 were higher in the combined PFT-α and UA treated groups than UA treatment groups. In addition, the p53 expression level in the PFT-α combined with UA groups were observed to decrease after adding PFT-α. These results indicated that UA may decrease the expression levels of PLK1 and CCNB1 and induce the apoptosis of MDA-MB-231 and MDA-MB-468 cells through the p53 signaling pathway, which was consistent with the results of the above-mentioned comprehensive bioinformatics analysis.

### Effect of UA on the Tumor in Xenograft Mice

**FIGURE 13:**
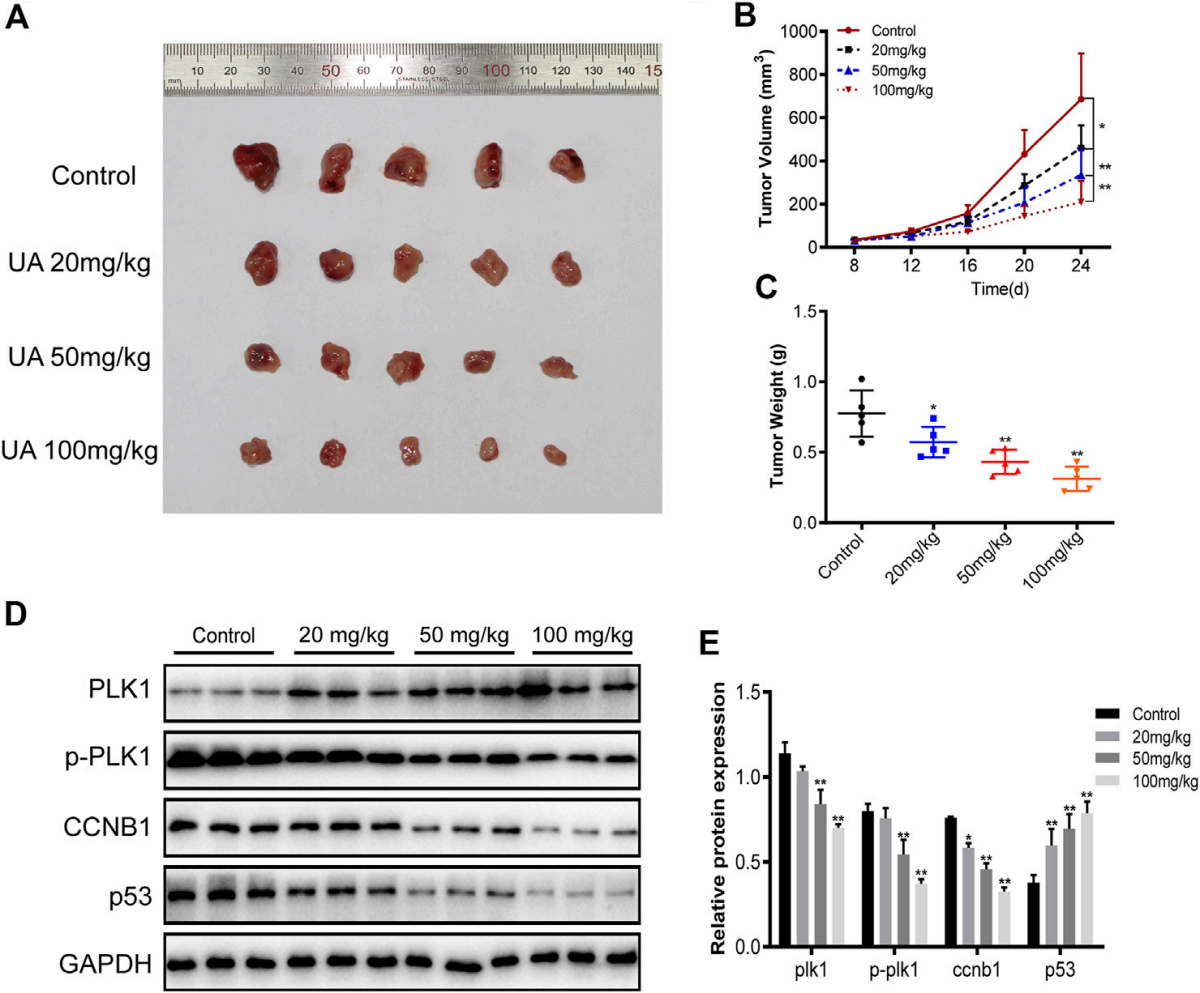
The anti-TNBC activity of UA was evaluated in xenograft mice. **(A)** The photo collection of xenograft subcutaneous tumors in nude mice. UA suppresses the *in vivo* tumor development of MDA-MB-231 cells. Compared with the control group, the tumor volume **(B)** and tumor weight **(C)** of xenograft tumors treated with UA were significantly reduced (*n* = 5 per group). **(D)** Western blot analyses were performed for PLK1, p-PLK1, CCNB1, and P53 protein expressions in xenograft tumors (*n* = 3 per group). Data are presented as the mean ± standard deviation (*n* = 3), *p < 0.05 and**p < 0.01 compared with control.

To evaluate the activity of UA against TNBC *in vivo*, the BALB/c female nude mice xenograft model was established in this study. As shown in Figures 13A,C, the tumor volume of the control xenografts was 686.6 ± 211.9 mm^3^ and tumor weight of 0.7760 ± 0.1647 g. The subcutaneous tumor volume of nude mice in the UA-treated xenografts was significantly reduced. The tumor volume was reduced to 460.7 ± 103.4 mm^3^ (20 mg/kg), 335.4 ± 123.0 mm^3^ (50 mg/kg), and 209.5 ± 97.90 mm^3^ (100 mg/kg). While tumor weight was reduced to 0.5720 ± 0.11, 0.4320 ± 0.09, and 0.3120 ± 0.09 g. Western blot analysis was shown in Figures 13D,E. The protein expression levels of PLK1, p-PLK1, and CCNB1 in the UA-treated xenografts were significantly reduced, compared with the control xenografts. Meanwhile, the protein expression level of p53 in the UA-treated xenografts was significantly increased when compared with the control xenografts.

## Discussion

As a disease with complex heterogeneity, TNBC not only lacks the expression of estrogen receptor, progesterone receptor and human epidermal growth factor receptor-2 at hormone level, but also shows more diversity at molecular genetic level compared with other subtypes of breast cancer (Kalimutho et al., 2015). The molecular biology map of TNBC shows that its occurrence and development is a multi-level evolutionary process, which is also the main reason for TNBC’s prone to relapse, metastasis and lack of treatment strategies (Hwang et al., 2019).

The obvious advantage of natural products over conventional chemotherapy is that they could interact with multiple targets, and on account of their high therapeutic value and low systemic toxicity, they have attracted widespread attention as emerging anticancer agents (Rahman, 2016; Yarla et al., 2016). UA exhibits strongly pharmacological activity and has been reported to be an effective anti-inflammatory and antiviral molecule, regulator of immune function, and anti-tumor agent (Hussain et al., 2017) in carcinomas, such as colorectal carcinoma (Kim et al., 2018), hepatocellular carcinoma (Zhou et al., 2019), and non-small-cell lung carcinoma (Yang et al., 2019). In addition, UA has been shown to reverse the resistance of TNBC to doxorubicin and paclitaxel in clinical treatment.

In the present study, UA was proven to significantly inhibit the proliferation of MDA-MB-231 and MDA-MB-468 cell lines through EDU staining experiments. It is worth noting that UA showed lower cytotoxic to human normal breast epithelial HMEC cells compared with tumor cells. Meanwhile, we also found that UA could block DNA production and induce arresting in G2/M phase in cell cycle and induce apoptosis of MDA-MB-231 and MDA-MB-468 cells, suggesting that UA holds plentiful pharmacological activity against TNBC. These findings indicated that UA has broadly prospects in clinical application. Furthermore, bioinformatics and molecular docking were performed to study the molecular mechanism of UA against TNBC. GSEA identified the critical signaling pathways of the targets to explore the mechanism of UA intervention in TNBC. The results indicated that UA may affect the expression of PLK1 and CCNB1 through regulation of the p53 signaling pathway. It is worth noting that critical pathways are highly correlated with cancer progression. The results of *in vivo* experiments were basically consistent with those of *in vitro* experiments. Compared with the control group, tumors growth in the UA treated xenografts were significantly slower in the medication group. Thus, we considered that the anti-TNBC effect of UA deserves recognition.

TNBC has worse prognosis, higher proliferation and metastasis rate, and usually appears in the form of highly invasive ductal carcinoma, compared with other breast cancer subtypes (Kemp et al., 2015). The fact that the median OS of patients with metastatic TNBC is 18 months emphasizes the importance of elucidating the molecular drivers of TNBC metastasis (Lehmann et al., 2011). Cancer metastasis is complex and regulated by many factors, since metastasis starts with the infiltration of the original tumor tissue, cancer cells often gain migration and invasion capabilities by changing gene expression during the epithelial to mesenchymal transition (EMT) process (Shin et al., 2020). PLK1 is a key regulator of proto-oncogenes and several cellular events, including cell division, DNA replication, and DNA damage repair (Barr et al., 2004; Yoo et al., 2004). Its expression is up-regulated in many invasive cancers, including breast (Wolf et al., 2000), gastric (Tokumitsu et al., 1999) and colon cancer (Takahashi et al., 2003). STAT3, an important member of the JAK/STAT signaling pathway, is negatively regulated or activated by PLK1, leading to the up-regulation of the downstream gene MMP2, thereby enhancing the aggressiveness of tumor cells (Yan et al., 2018). It has been reported that active PLK1 promotes metastasis by up-regulating TGF-β signaling and amplifies its metastatic properties by forming a positive feedback loop (Shin et al., 2020). In addition, PLK1 has also been reported to increase the invasiveness of vimentin-expressing cells by regulating the level of cell surface receptor β1 integrin (Lambert et al., 2017).

Intact apoptosis mechanisms are generally dysregulated in cancer (Degterev and Yuan, 2008). The importance of apoptosis disorders in the prognosis of TNBC has been fully demonstrated (Vagia et al., 2020). Caspase-8, a member of the caspase family of proteases, is a key driving factor for apoptosis (Degterev et al., 2003). Studies have shown that Cdk1 and cyclin B1 phosphorylate procaspase-8 at Ser-387, thereby inhibiting the activation of procaspase-8 (Matthess et al., 2010). Meanwhile, initiator caspases that act upstream in the apoptotic cascade are mediated by cdk1/cyclin B1 to block the apoptosis of mitotic cells (Allan and Clarke, 2007; Andersen et al., 2009).

The p53 gene is a well-known tumor suppressor and an important factor in tumor progression (Machado-Silva et al., 2010). According to statistics, the inactivation rate of p53 in various types of tumors exceeds 50% and reaches 75% in invasive cancers (Golubovskaya and Cance, 2013). The p53 transcription factor is induced in response to DNA damage, hypoxia, and oncogene activation (Ventura et al., 2007), and loss of p53 activity indicates poor prognosis, as it regulates the gene expression program leading to cell cycle arrest and apoptosis (Sherr and McCormick, 2002). Some scholars support the view that restoring the p53 pathway could effectively treat breast cancer (Moulder et al., 2018). Studies have shown that the function of p53 was restored after inhibiting CCNB1/Cdk1, which suggests that CCNB1 is involved in inactivating p53 function and promoting tumorigenesis (Lu et al., 2013). Based on this, PLK1 and CCNB1 may be key factors in the mechanism of UA intervention in TNBC.

In this study, we reported that UA could down-regulate the expression levels of MMP2 and MMP9 to mediate the migration and invasion of TNBC cells. At the same time, it was also found that UA could induce TNBC cell apoptosis by down-regulating the expression of Bcl-2 and increasing the expression levels of cleaved caspase-3 and cleaved caspase-9. qRT-PCR and western blot analysis showed that UA significantly reduced the mRNA and protein expression levels of PLK1 and CCNB1 in MDA-MB-231 and MDA-MB-468 cells. Meanwhile, the expression level of p53 was significantly increased after UA treatment. These results reflected the potential pharmacological role of UA in the treatment of TNBC.

In summary, biomarkers closely related to TNBC were screened, and *in vitro* experiments showed that UA could significantly inhibit the proliferation, migration, invasion and induce cell cycle arrest and apoptosis of MDA-MB-231 and MDA-MB-468 cells with a concentration-dependent manner. After UA treatment, the tumor body and tumor weight of TNBC-xenograft mice decreased significantly, indicating that it could inhibit tumor growth *in vivo*. It suggests that the potential mechanism for UA against TNBC was to down-regulate the protein expression levels of PLK1 and CCNB1 through the p53 signaling pathway. Furthermore, this study combines bioinformatics and experimental verification to provide a new perspective for studying the multi-target effects of natural small-molecule compounds in treating malignancies.

## Data Availability

Publicly available datasets were analyzed in this study, these can be found in the NCBI Gene Expression Omnibus (GSE45827, GSE65194).

## References

[B1] Allan L. A. Clarke P. R. (2007). Phosphorylation of Caspase-9 by CDK1/cyclin B1 Protects Mitotic Cells against Apoptosis. Mol. Cel 26 (2), 301–310. 10.1016/j.molcel.2007.03.019 17466630

[B2] Alonso H. Bliznyuk A. A. Gready J. E. (2006). Combining Docking and Molecular Dynamic Simulations in Drug Design. Med. Res. Rev. 26 (5), 531–568. 10.1002/med.20067 16758486

[B3] Andersen J. L. Johnson C. E. Freel C. D. Parrish A. B. Day J. L. Buchakjian M. R. (2009). Restraint of Apoptosis during Mitosis through Interdomain Phosphorylation of Caspase-2. EMBO J. 28 (20), 3216–3227. 10.1038/emboj.2009.253 19730412 PMC2771089

[B4] Barr F. A. Silljé H. H. Nigg E. A. (2004). Polo-like Kinases and the Orchestration of Cell Division. Nat. Rev. Mol. Cel Biol 5 (6), 429–440. 10.1038/nrm1401 15173822

[B5] Casado P. Zuazua-Villar P. del Valle E. Martínez-Campa C. Lazo P. S. Ramos S. (2007). Vincristine Regulates the Phosphorylation of the Antiapoptotic Protein HSP27 in Breast Cancer Cells. Cancer Lett. 247 (2), 273–282. 10.1016/j.canlet.2006.05.005 16843591

[B6] Chan E. W. C. Soon C. Y. Tan J. B. L. Wong S. K. Hui Y. W. (2019). Ursolic Acid: An Overview on its Cytotoxic Activities against Breast and Colorectal Cancer Cells. J. Integr. Med. 17 (3), 155–160. 10.1016/j.joim.2019.03.003 30928277

[B7] Degterev A. Boyce M. Yuan J. (2003). A Decade of Caspases. Oncogene 22 (53), 8543–8567. 10.1038/sj.onc.1207107 14634618

[B8] Degterev A. Yuan J. (2008). Expansion and Evolution of Cell Death Programmes. Nat. Rev. Mol. Cel Biol 9 (5), 378–390. 10.1038/nrm2393 18414491

[B9] DeSantis C. E. Ma J. Gaudet M. M. Newman L. A. Miller K. D. Goding Sauer A. (2019). Breast Cancer Statistics, 2019. CA Cancer J. Clin. 69 (6), 438–451. 10.3322/caac.21583 31577379

[B10] Gluz O. Nitz U. Liedtke C. Christgen M. Grischke E. M. Forstbauer H. (2018). Comparison of Neoadjuvant Nab-Paclitaxel+Carboplatin vs Nab-Paclitaxel+Gemcitabine in Triple-Negative Breast Cancer: Randomized WSG-ADAPT-TN Trial Results. J. Natl. Cancer Inst. 110 (6), 628–637. 10.1093/jnci/djx258 29228315

[B11] Golubovskaya V. M. Cance W. G. (2013). Targeting the P53 Pathway. Surg. Oncol. Clin. N. Am. 22 (4), 747–764. 10.1016/j.soc.2013.06.003 24012397 PMC3810242

[B12] Gruosso T. Mieulet V. Cardon M. Bourachot B. Kieffer Y. Devun F. (2016). Chronic Oxidative Stress Promotes H2AX Protein Degradation and Enhances Chemosensitivity in Breast Cancer Patients. EMBO Mol. Med. 8 (5), 527–549. 10.15252/emmm.201505891 27006338 PMC5123617

[B13] Hopkins A. L. (2008). Network Pharmacology: the Next Paradigm in Drug Discovery. Nat. Chem. Biol. 4 (11), 682–690. 10.1038/nchembio.118 18936753

[B14] Huang C. Y. Lin C. Y. Tsai C. W. Yin M. C. (2011). Inhibition of Cell Proliferation, Invasion and Migration by Ursolic Acid in Human Lung Cancer Cell Lines. Toxicol. Vitro 25 (7), 1274–1280. 10.1016/j.tiv.2011.04.014 21539908

[B15] Huang D. W. Sherman B. T. Tan Q. Kir J. Liu D. Bryant D. (2007). DAVID Bioinformatics Resources: Expanded Annotation Database and Novel Algorithms to Better Extract Biology from Large Gene Lists. Nucleic Acids Res. 35, W169–W175. 10.1093/nar/gkm415 17576678 PMC1933169

[B16] Huang M. Lu J. J. Huang M. Q. Bao J. L. Chen X. P. Wang Y. T. (2012). Terpenoids: Natural Products for Cancer Therapy. Expert Opin. Investig. Drugs 21 (12), 1801–1818. 10.1517/13543784.2012.727395 23092199

[B17] Hussain H. Green I. R. Ali I. Khan I. A. Ali Z. Al-Sadi A. M. (2017). Ursolic Acid Derivatives for Pharmaceutical Use: a Patent Review (2012-2016). Expert Opin. Ther. Pat 27 (9), 1061–1072. 10.1080/13543776.2017.1344219 28637397

[B18] Hwang S. Y. Park S. Kwon Y. (2019). Recent Therapeutic Trends and Promising Targets in Triple Negative Breast Cancer. Pharmacol. Ther. 199, 30–57. 10.1016/j.pharmthera.2019.02.006 30825473

[B19] Ingvarsson S. (2017). Molecular Genetics of Breast Cancer. Int. J. Hum. Genet. 3 (2), 69–78. 10.1080/09723757.2003.11885831

[B20] Israel B. B. Tilghman S. L. Parker-Lemieux K. Payton-Stewart F. (2018). Phytochemicals: Current Strategies for Treating Breast Cancer. Oncol. Lett. 15 (5), 7471–7478. 10.3892/ol.2018.8304 29755596 PMC5943681

[B21] Kalimutho M. Parsons K. Mittal D. López J. A. Srihari S. Khanna K. K. (2015). Targeted Therapies for Triple-Negative Breast Cancer: Combating a Stubborn Disease. Trends Pharmacol. Sci. 36 (12), 822–846. 10.1016/j.tips.2015.08.009 26538316

[B22] Kemp T. L. Kilgore M. R. Javid S. H. (2015). Invasive Ductal Carcinoma Arising within a Large Mammary Hamartoma. Breast J. 21 (2), 196–197. 10.1111/tbj.12378 25613435

[B23] Kim K. Shin E. A. Jung J. H. Park J. E. Kim D. S. Shim B. S. (2018). Ursolic Acid Induces Apoptosis in Colorectal Cancer Cells Partially via Upregulation of MicroRNA-4500 and Inhibition of JAK2/STAT3 Phosphorylation. Int. J. Mol. Sci. 20 (1). 10.3390/ijms20010114 PMC6337206 30597956

[B24] Kim S. Chen J. Cheng T. Gindulyte A. He J. He S. (2019). PubChem 2019 Update: Improved Access to Chemical Data. Nucleic Acids Res. 47 (D1), D1102–D1109. 10.1093/nar/gky1033 30371825 PMC6324075

[B25] Lambert A. W. Pattabiraman D. R. Weinberg R. A. (2017). Emerging Biological Principles of Metastasis. Cell 168 (4), 670–691. 10.1016/j.cell.2016.11.037 28187288 PMC5308465

[B26] Lehmann B. D. Bauer J. A. Chen X. Sanders M. E. Chakravarthy A. B. Shyr Y. (2011). Identification of Human Triple-Negative Breast Cancer Subtypes and Preclinical Models for Selection of Targeted Therapies. J. Clin. Invest. 121 (7), 2750–2767. 10.1172/JCI45014 21633166 PMC3127435

[B27] Li S. Fan T. P. Jia W. Lu A. Zhang W. (2014). Network Pharmacology in Traditional Chinese Medicine. Evid. Based Complement. Alternat Med. 2014, 138460. 10.1155/2014/138460 24707305 PMC3953584

[B28] Lin C. C. Huang C. Y. Mong M. C. Chan C. Y. Yin M. C. (2011). Antiangiogenic Potential of Three Triterpenic Acids in Human Liver Cancer Cells. J. Agric. Food Chem. 59 (2), 755–762. 10.1021/jf103904b 21175131

[B29] Liu T. Ma H. Shi W. Duan J. Wang Y. Zhang C. (2017). Inhibition of STAT3 Signaling Pathway by Ursolic Acid Suppresses Growth of Hepatocellular Carcinoma. Int. J. Oncol. 51 (2), 555–562. 10.3892/ijo.2017.4035 28714512

[B30] Lu M. Breyssens H. Salter V. Zhong S. Hu Y. Baer C. (2013). Restoring P53 Function in Human Melanoma Cells by Inhibiting MDM2 and Cyclin B1/CDK1-Phosphorylated Nuclear iASPP. Cancer Cell 23 (5), 618–633. 10.1016/j.ccr.2013.03.013 23623661

[B31] Lu Q. Chen W. Ji Y. Liu Y. Xue X. (2021). Ursolic Acid Enhances Cytotoxicity of Doxorubicin-Resistant Triple-Negative Breast Cancer Cells via ZEB1-AS1/miR-186-5p/ABCC1 Axis. Cancer Biother. Radiopharm. 10.1089/cbr.2020.4147 33493421

[B32] Lv X. He C. Huang C. Hua G. Wang Z. Remmenga S. W. (2017). G-1 Inhibits Breast Cancer Cell Growth via Targeting Colchicine-Binding Site of Tubulin to Interfere with Microtubule Assembly. Mol. Cancer Ther. 16 (6), 1080–1091. 10.1158/1535-7163.MCT-16-0626 28258163 PMC5457708

[B33] Machado-Silva A. Perrier S. Bourdon J. C. (2010). p53 Family Members in Cancer Diagnosis and Treatment. Semin. Cancer Biol. 20 (1), 57–62. 10.1016/j.semcancer.2010.02.005 20206267

[B34] Maire V. Némati F. Richardson M. Vincent-Salomon A. Tesson B. Rigaill G. (2013). Polo-like Kinase 1: a Potential Therapeutic Option in Combination with Conventional Chemotherapy for the Management of Patients with Triple-Negative Breast Cancer. Cancer Res. 73 (2), 813–823. 10.1158/0008-5472.CAN-12-2633 23144294

[B35] Manayi A. Nikan M. Nobakht-Haghighi N. Abdollahi M. (2018). Advances in the Anticancer Value of the Ursolic Acid through Nanodelivery. Curr. Med. Chem. 25 (37), 4866–4875. 10.2174/0929867324666170713102918 28707589

[B36] Matthess Y. Raab M. Sanhaji M. Lavrik I. N. Strebhardt K. (2010). Cdk1/cyclin B1 Controls Fas-Mediated Apoptosis by Regulating Caspase-8 Activity. Mol. Cel Biol 30 (24), 5726–5740. 10.1128/MCB.00731-10 PMC3004266 20937773

[B37] Moulder D. E. Hatoum D. Tay E. Lin Y. McGowan E. M. (2018). The Roles of P53 in Mitochondrial Dynamics and Cancer Metabolism: The Pendulum between Survival and Death in Breast Cancer? Cancers (Basel) 10 (6). 10.3390/cancers10060189 PMC6024909 29890631

[B38] Nedeljković M. Damjanović A. (2019). Mechanisms of Chemotherapy Resistance in Triple-Negative Breast Cancer-How We Can Rise to the Challenge. Cells 8 (9), 957. 10.3390/cells8090957 PMC6770896 31443516

[B39] Oulas A. Minadakis G. Zachariou M. Sokratous K. Bourdakou M. M. Spyrou G. M. (2019). Systems Bioinformatics: Increasing Precision of Computational Diagnostics and Therapeutics through Network-Based Approaches. Brief Bioinform 20 (3), 806–824. 10.1093/bib/bbx151 29186305 PMC6585387

[B40] Pavelić K. Gall-Trošelj K. (2001). Recent Advances in Molecular Genetics of Breast Cancer. J. Mol. Med. 79 (10), 566–573. 10.1007/s001090100256 11692153

[B41] Rahman H. S. (2016). Natural Products for Cancer Therapy. Dual Diagn. Open Acc. 1 (3). 10.21767/2472-5048.100015

[B42] Rubovszky G. Horváth Z. (2017). Recent Advances in the Neoadjuvant Treatment of Breast Cancer. J. Breast Cancer 20 (2), 119–131. 10.4048/jbc.2017.20.2.119 28690648 PMC5500395

[B43] Shannon P. Markiel A. Ozier O. Baliga N. S. Wang J. T. Ramage D. (2003). Cytoscape: a Software Environment for Integrated Models of Biomolecular Interaction Networks. Genome Res. 13 (11), 2498–2504. 10.1101/gr.1239303 14597658 PMC403769

[B44] Sharma P. (2016). Biology and Management of Patients with Triple-Negative Breast Cancer. Oncologist 21 (9), 1050–1062. 10.1634/theoncologist.2016-0067 27401886 PMC5016071

[B45] Sherr C. J. McCormick F. (2002). The RB and P53 Pathways in Cancer. Cancer cell 2 (2), 103–112. 10.1016/s1535-6108(02)00102-2 12204530

[B46] Shin S. B. Jang H. R. Xu R. Won J. Y. Yim H. (2020). Active PLK1-Driven Metastasis Is Amplified by TGF-β Signaling that Forms a Positive Feedback Loop in Non-small Cell Lung Cancer. Oncogene 39 (4), 767–785. 10.1038/s41388-019-1023-z 31548612 PMC6976524

[B47] Stratton M. R. Campbell P. J. Futreal P. A. (2009). The Cancer Genome. Nature 458 (7239), 719–724. 10.1038/nature07943 19360079 PMC2821689

[B48] Szklarczyk D. Franceschini A. Wyder S. Forslund K. Heller D. Huerta-Cepas J. (2015). STRING V10: Protein-Protein Interaction Networks, Integrated over the Tree of Life. Nucleic Acids Res. 43 (Database issue), D447–D452. 10.1093/nar/gku1003 25352553 PMC4383874

[B49] Szklarczyk D. Morris J. H. Cook H. Kuhn M. Wyder S. Simonovic M. (2017). The STRING Database in 2017: Quality-Controlled Protein-Protein Association Networks, Made Broadly Accessible. Nucleic Acids Res. 45 (D1), D362–D368. 10.1093/nar/gkw937 27924014 PMC5210637

[B50] Takahashi T. Sano B. Nagata T. Kato H. Sugiyama Y. Kunieda K. (2003). Polo-like Kinase 1 (PLK1) Is Overexpressed in Primary Colorectal Cancers. Cancer Sci. 94 (2), 148–152. 10.1111/j.1349-7006.2003.tb01411.x 12708489 PMC11160284

[B51] Tang Z. Li C. Kang B. Gao G. Li C. Zhang Z. (2017). GEPIA: a Web Server for Cancer and normal Gene Expression Profiling and Interactive Analyses. Nucleic Acids Res. 45 (W1), W98–W102. 10.1093/nar/gkx247 28407145 PMC5570223

[B52] Tokumitsu Y. Mori M. Tanaka S. Akazawa K. Nakano S. Niho Y. (1999). Prognostic Significance of polo-like Kinase Expression in Esophageal Carcinoma. Int. J. Oncol. 15 (4), 687–692. 10.3892/ijo.15.4.687 10493949

[B53] Vagia E. Mahalingam D. Cristofanilli M. (2020). The Landscape of Targeted Therapies in TNBC. Cancers (Basel) 12 (4). 10.3390/cancers12040916 PMC7226210 32276534

[B54] Ventura A. Kirsch D. G. McLaughlin M. E. Tuveson D. A. Grimm J. Lintault L. (2007). Restoration of P53 Function Leads to Tumour Regression *In Vivo* . Nature 445 (7128), 661–665. 10.1038/nature05541 17251932

[B55] Wang S. Meng X. Dong Y. (2017). Ursolic Acid Nanoparticles Inhibit Cervical Cancer Growth *In Vitro* and *In Vivo* via Apoptosis Induction. Int. J. Oncol. 50 (4), 1330–1340. 10.3892/ijo.2017.3890 28259944

[B56] Wolf G. Hildenbrand R. Schwar C. Grobholz R. Kaufmann M. Stutte H. J. (2000). Polo-like Kinase: a Novel Marker of Proliferation: Correlation with Estrogen-Receptor Expression in Human Breast Cancer. Pathol. Res. Pract. 196 (11), 753–759. 10.1016/s0344-0338(00)80107-7 11186170

[B57] Xiang F. Fan Y. Ni Z. Liu Q. Zhu Z. Chen Z. (2019). Ursolic Acid Reverses the Chemoresistance of Breast Cancer Cells to Paclitaxel by Targeting MiRNA-149-5p/MyD88. Front. Oncol. 9, 501. 10.3389/fonc.2019.00501 31259152 PMC6587017

[B58] Yan W. Yu H. Li W. Li F. Wang S. Yu N. (2018). Plk1 Promotes the Migration of Human Lung Adenocarcinoma Epithelial Cells via STAT3 Signaling. Oncol. Lett. 16 (5), 6801–6807. 10.3892/ol.2018.9437 30405824 PMC6202555

[B59] Yang K. Chen Y. Zhou J. Ma L. Shan Y. Cheng X. (2019). Ursolic Acid Promotes Apoptosis and Mediates Transcriptional Suppression of CT45A2 Gene Expression in Non-small-cell Lung Carcinoma Harbouring EGFR T790M Mutations. Br. J. Pharmacol. 176 (24), 4609–4624. 10.1111/bph.14793 31322286 PMC6965687

[B60] Yarla N. S. Bishayee A. Sethi G. Reddanna P. Kalle A. M. Dhananjaya B. L. (2016). Targeting Arachidonic Acid Pathway by Natural Products for Cancer Prevention and Therapy. Semin. Cancer Biol. 40-41, 48–81. 10.1016/j.semcancer.2016.02.001 26853158

[B61] Yin R. Li T. Tian J. X. Xi P. Liu R. H. (2018). Ursolic Acid, a Potential Anticancer Compound for Breast Cancer Therapy. Crit. Rev. Food Sci. Nutr. 58 (4), 568–574. 10.1080/10408398.2016.1203755 27469428

[B62] Yoo H. Y. Kumagai A. Shevchenko A. Shevchenko A. Dunphy W. G. (2004). Adaptation of a DNA Replication Checkpoint Response Depends upon Inactivation of Claspin by the Polo-like Kinase. Cell 117 (5), 575–588. 10.1016/s0092-8674(04)00417-9 15163406

[B63] Zhou M. Yi Y. Liu L. Lin Y. Li J. Ruan J. (2019). Polymeric Micelles Loading with Ursolic Acid Enhancing Anti-tumor Effect on Hepatocellular Carcinoma. J. Cancer 10 (23), 5820–5831. 10.7150/jca.30865 31737119 PMC6843872

