# Supplementary material for: Identifying the Effect of Ursolic Acid Against Triple-Negative Breast Cancer: Coupling Network Pharmacology With Experiments Verification
Source: Front Pharmacol. 2021 Nov 11;12:685773. doi: 10.3389/fphar.2021.685773 (PMC8631906; doi:10.3389/fphar.2021.685773)

1. CCK-8

concentrati	24h					48h					72h				
Control	1.365	1.368	1.439	1.263	1.253	1.37	1.37	1.458	1.424	1.307	1.241	1.26	1.394	1.236	1.366
DMSO	1.365	1.398	1.422	1.427	1.491	1.331	1.424	1.347	1.367	1.463	1.325	1.381	1.378	1.366	1.452
15uM	1.145	1.092	1.133	1.166	1.104	1.051	0.997	0.972	0.96	1.07	0.866	0.911	0.916	0.902	0.98
20uM	1.078	1.093	1.002	1.047	1.068	0.684	0.689	0.741	0.699	0.747	0.577	0.573	0.602	0.601	0.56
25uM	0.831	0.845	0.809	0.808	0.837	0.517	0.425	0.436	0.417	0.509	0.462	0.441	0.45	0.434	0.436
30uM	0.706	0.724	0.738	0.721	0.714	0.449	0.409	0.425	0.4	0.436	0.337	0.354	0.337	0.322	0.367
blank	0.046	0.048	0.047	0.047	0.049										
concentrati							Relative survival rate								
Control	1	1.002276	1.056146	0.92261	0.915023	1.003794	1.003794	1.070561	1.044765	0.955994	0.905918	0.920334	1.022003	0.902124	1.000759
DMSO	1	1.025038	1.043247	1.047041	1.095599	0.974203	1.044765	0.986343	1.001517	1.074355	0.969651	1.01214	1.009863	1.000759	1.066009
15uM	0.83308	0.792868	0.823976	0.849014	0.801973	0.76176	0.720789	0.701821	0.692716	0.776176	0.621396	0.655539	0.659332	0.64871	0.707891
20uM	0.782246	0.793627	0.724583	0.758725	0.774659	0.483308	0.487102	0.526555	0.494689	0.531108	0.402124	0.39909	0.421093	0.420334	0.389226
25uM	0.594841	0.605463	0.578149	0.57739	0.599393	0.356601	0.286798	0.295144	0.280728	0.350531	0.314871	0.298938	0.305766	0.293627	0.295144
30uM	0.5	0.513657	0.524279	0.511381	0.50607	0.305008	0.274659	0.286798	0.26783	0.295144	0.22003	0.232929	0.22003	0.208649	0.242792
MDA-MB-231															

concentrati	24h					48h					72h				
Control	1.302	1.305	1.383	1.189	1.178	1.307	1.307	1.404	1.366	1.238	1.265	1.186	1.333	1.216	1.303
DMSO	1.302	1.338	1.364	1.37	1.44	1.264	1.366	1.282	1.304	1.409	1.258	1.319	1.316	1.303	1.397
15uM	1.206	1.101	1.146	1.183	1.114	0.956	0.897	0.869	0.856	0.977	1.153	1.202	1.208	1.192	1.078
20uM	0.986	1.002	0.902	0.952	0.975	0.802	0.808	0.865	0.819	0.872	0.935	0.93	0.902	0.901	0.916
25uM	0.814	0.83	0.79	0.789	0.821	0.769	0.668	0.68	0.659	0.76	0.608	0.585	0.595	0.577	0.58
30uM	0.677	0.696	0.712	0.693	0.685	0.714	0.67	0.688	0.66	0.7	0.471	0.489	0.471	0.454	0.504
blank	0.045	0.044	0.045	0.047	0.046										
concentrati							Relative survival rate								
Control	1	1.002387	1.064439	0.910103	0.901352	1.003978	1.003978	1.081146	1.050915	0.949085	0.970565	0.907717	1.024662	0.931583	1.000796
DMSO	1	1.02864	1.049324	1.054097	1.109785	0.969769	1.050915	0.984089	1.001591	1.085123	0.964996	1.013524	1.011138	1.000796	1.075577
15uM	0.923628	0.840095	0.875895	0.90533	0.850438	0.724741	0.677804	0.655529	0.645187	0.741448	0.881464	0.920446	0.925219	0.91249	0.821798
20uM	0.748608	0.761337	0.681782	0.721559	0.739857	0.602228	0.607001	0.652347	0.615752	0.657916	0.708035	0.704057	0.681782	0.680986	0.69292
25uM	0.611774	0.624503	0.592681	0.591885	0.617343	0.575975	0.495625	0.505171	0.488465	0.568815	0.447892	0.429594	0.43755	0.42323	0.425617
30uM	0.502784	0.5179	0.530628	0.515513	0.509149	0.53222	0.497216	0.511535	0.48926	0.521082	0.338902	0.353222	0.338902	0.325378	0.365155
MDA-MB-468															

concentrati	24h					48h					72h				
Control	1.394	1.336	1.477	1.494	1.48	1.422	1.476	1.47	1.597	1.555	1.484	1.372	1.333	1.476	1.466
DMSO	1.338	1.477	1.378	1.555	1.491	1.21	1.209	1.243	1.222	1.191	1.436	1.469	1.474	1.463	1.351
15uM	1.424	1.419	1.384	1.447	1.379	1.2	1.239	1.239	1.239	1.178	1.319	1.303	1.354	1.303	1.35
20uM	1.319	1.388	1.423	1.44	1.34	0.97	0.993	0.977	0.937	0.997	1.177	1.163	1.191	1.142	1.07
25uM	1.283	1.254	1.289	1.199	1.178	0.797	0.774	0.721	0.809	0.707	0.969	0.943	0.894	0.934	0.848
30uM	1.059	1.099	1.102	1.095	1.12	0.69	0.569	0.558	0.621	0.639	0.66	0.624	0.667	0.74	0.763
blank	0.046	0.047	0.045	0.048	0.046										
concentrati							Relative survival rate								
Control	1.000742	0.957715	1.062315	1.074926	1.06454	1.021513	1.061573	1.057122	1.151335	1.120178	1.067507	0.984421	0.95549	1.061573	1.054154
DMSO	0.959199	1.062315	0.988872	1.120178	1.0727	0.864243	0.863501	0.888724	0.873145	0.850148	1.031899	1.05638	1.060089	1.051929	0.968843
15uM	1.022997	1.019288	0.993323	1.040059	0.989614	0.856825	0.885757	0.885757	0.885757	0.840504	0.945104	0.933234	0.971068	0.933234	0.968101
20uM	0.945104	0.996291	1.022255	1.034866	0.960682	0.686202	0.703264	0.691395	0.661721	0.706231	0.839763	0.829377	0.850148	0.813798	0.760386
25uM	0.918398	0.896884	0.922849	0.856083	0.840504	0.557864	0.540801	0.501484	0.566766	0.491098	0.68546	0.666172	0.629822	0.659496	0.595697
30uM	0.752226	0.781899	0.784125	0.778932	0.797478	0.478487	0.388724	0.380564	0.4273	0.440653	0.456231	0.429525	0.461424	0.515579	0.532641
HMEC															

2. EDU assay

MDA-MB-231

control

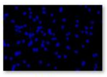
1 (1).jpg

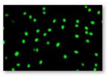
1 (2).jpg

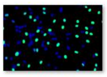
1 (3).jpg

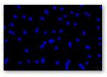
2 (1).jpg

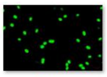
2 (2).jpg

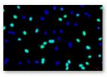
2 (3).jpg

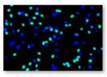
3 (1).jpg

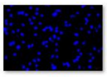
3 (2).jpg

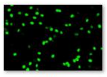
3 (3).jpg

DMSO

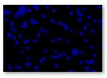
1 (1).jpg

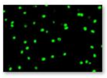
1 (2).jpg

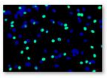
1 (3).jpg

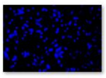
4 (1).jpg

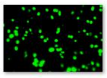
4 (2).jpg

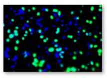
4 (3).jpg

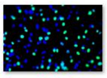
5 (1).jpg

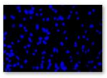
5 (2).jpg

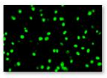
5 (3).jpg

15μM

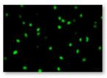
1 (1).jpg

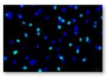
1 (2).jpg

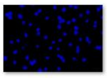
1 (3).jpg

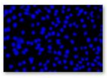
2 (1).jpg

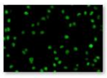
2 (2).jpg

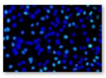
2 (3).jpg

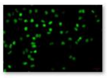
3 (1).jpg

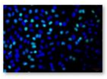
3 (2).jpg

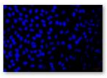
3 (3).jpg

20μM

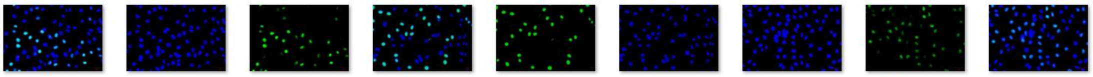

1 (1).jpg 1 (2).jpg 1 (3).jpg 3 (1).jpg 3 (2).jpg 3 (3).jpg 4 (1).jpg 4 (2).jpg 4 (3).jpg

25μM

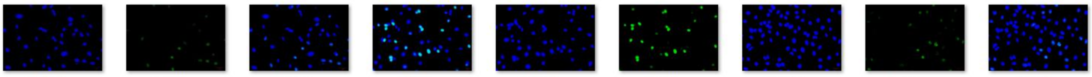

1 (1).jpg 1 (2).jpg 1 (3).jpg 3 (1).jpg 3 (2).jpg 3 (3).jpg 4 (1).jpg 4 (1).png 4 (2).jpg

MDA-MB-468

control

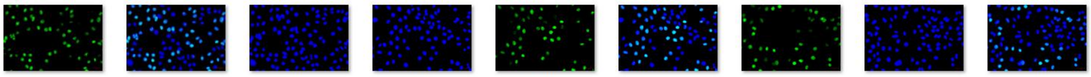

1 (1).jpg 1 (2).jpg 1 (3).jpg 2 (1).jpg 2 (2).jpg 2 (3).jpg 3 (1).jpg 3 (2).jpg 3 (3).jpg

DMSO

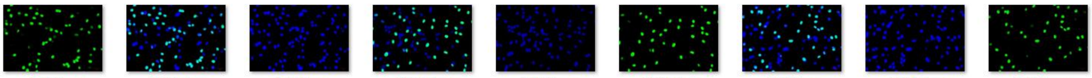

1 (1).jpg 1 (2).jpg 1 (3).jpg 2 (1).jpg 2 (2).jpg 2 (3).jpg 3 (1).jpg 3 (2).jpg 3 (3).jpg

15μM

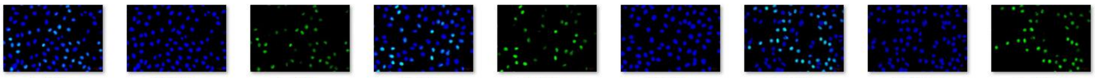

1 (1).jpg 1 (2).jpg 1 (3).jpg 2 (1).jpg 2 (2).jpg 2 (3).jpg 3 (1).jpg 3 (2).jpg 3 (3).jpg

20μM

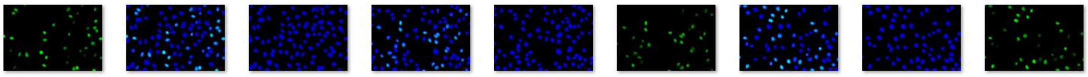

1 (1).jpg 1 (2).jpg 1 (3).jpg 2 (1).jpg 2 (2).jpg 2 (3).jpg 3 (1).jpg 3 (2).jpg 3 (3).jpg

25μM

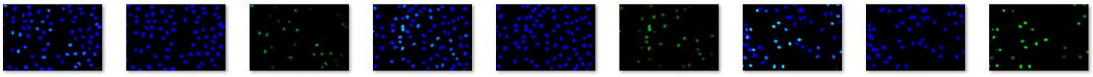

1 (1).jpg 1 (2).jpg 1 (3).jpg 2 (1).jpg 2 (2).jpg 2 (3).jpg 3 (1).jpg 3 (2).jpg 3 (3).jpg

3. Wound healing assay

MDA-MB-231

0h

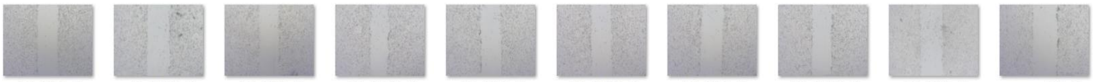

15μM(1).tif 15μM(2).tif 15μM(3).tif 20μM(1).tif 20μM(2).tif 20μM(3).tif 25μM(1).tif 25μM(2).tif 25μM(3).tif control(1).tif

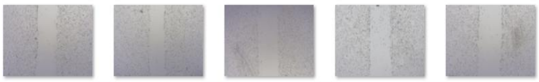

control(2).tif control(3).tif DMSO(1).tif DMSO(2).tif DMSO(3).tif

24h

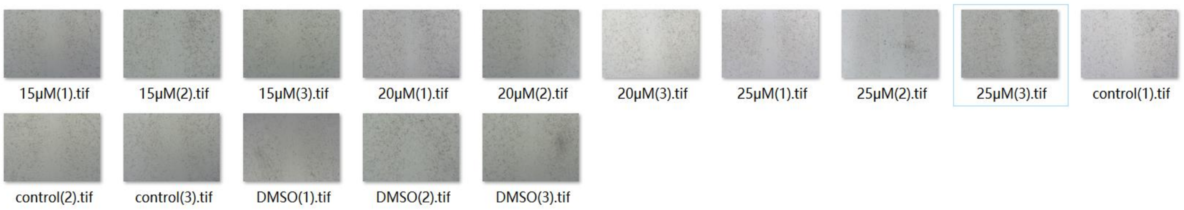

MDA-MB-468

(1)

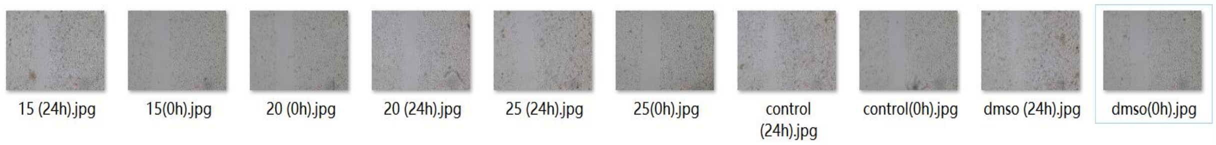

(2)

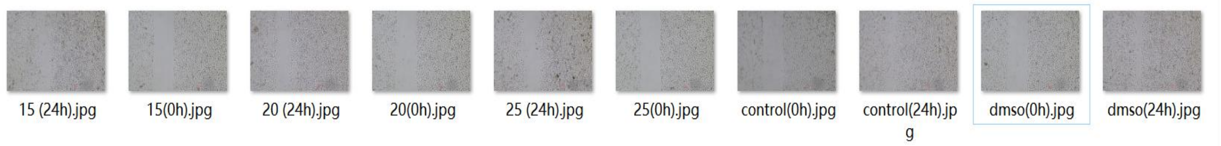

(3)

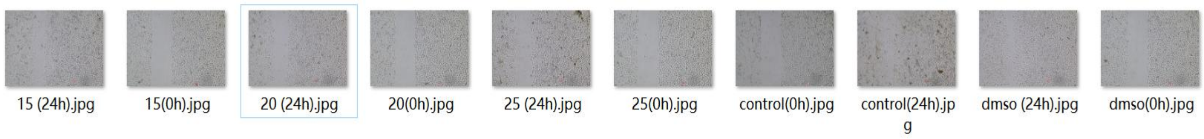

4. Invasion assay

MDA-MB-231

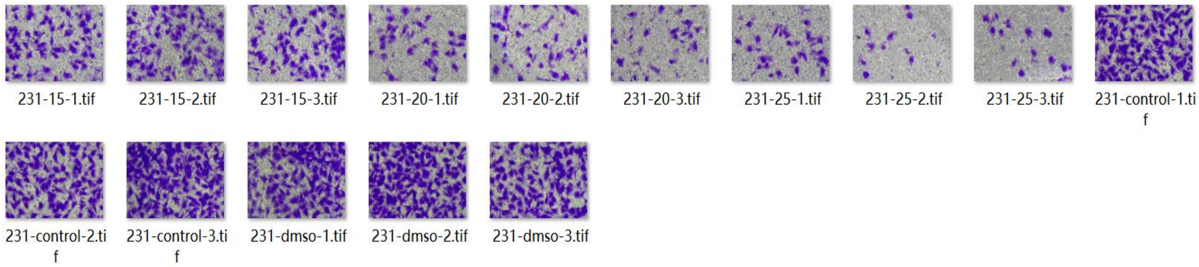

MDA-MB-468

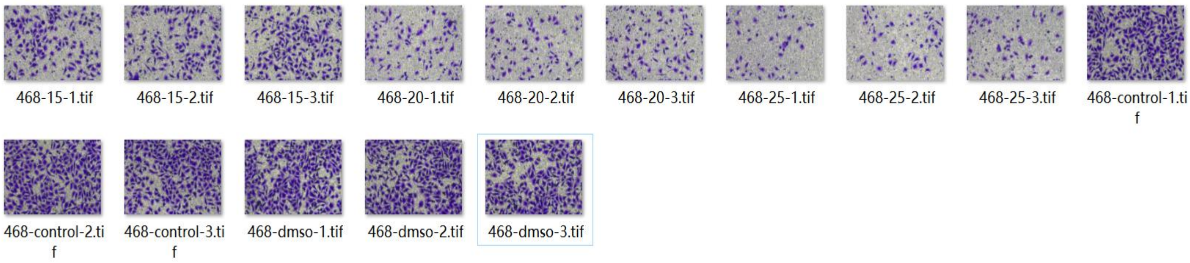

5. Apoptosis assay

MDA-MB-231

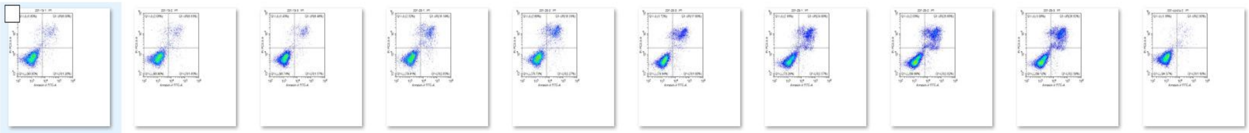

231-15-1_Plot1.bmp | 231-15-2_Plot1.bmp | 231-15-3_Plot1.bmp | 231-20-1_Plot1.bmp | 231-20-2_Plot1.bmp | 231-20-3_Plot1.bmp | 231-25-1_Plot1.bmp | 231-25-2_Plot1.bmp | 231-25-3_Plot1.bmp | 231-contro-2_Plot1.bmp

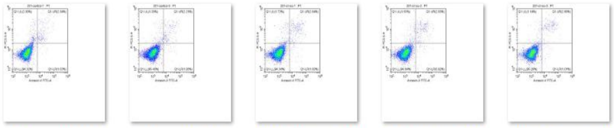

231-control-1_Plot1.bmp | 231-control-3_Plot1.bmp | 231-dmso-1_Plot1.bmp | 231-dmso-2_Plot1.bmp | 231-dmso-3_Plot1.bmp

MDA-MB-468

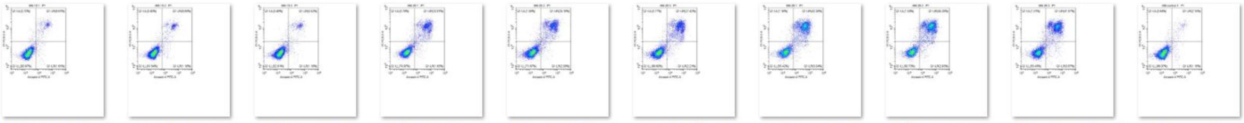

468-15-1_Plot1.bmp | 468-15-2_Plot1.bmp | 468-15-3_Plot1.bmp | 468-20-1_Plot1.bmp | 468-20-2_Plot1.bmp | 468-20-3_Plot1.bmp | 468-25-1_Plot1.bmp | 468-25-2_Plot1.bmp | 468-25-3_Plot1.bmp | 468-control-1_Plot1.bmp

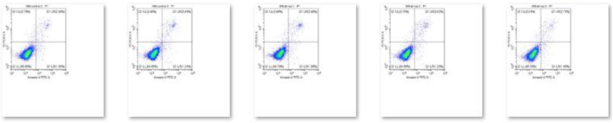

468-control-2_Plot1.bmp | 468-control-3_Plot1.bmp | 468-dmso-1_Plot1.bmp | 468-dmso-2_Plot1.bmp | 468-dmso-3_Plot1.bmp

6. Cell cycle assay

MDA-MB-231

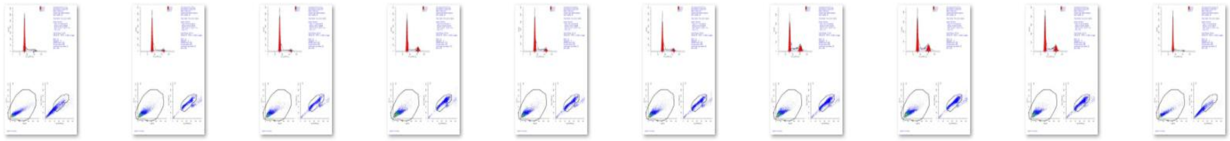

rpt_231-15-1.fcs.tif | rpt_231-15-2.fcs.tif | rpt_231-15-3.fcs.tif | rpt_231-20-1.fcs.tif | rpt_231-20-2.fcs.tif | rpt_231-20-3.fcs.tif | rpt_231-25-1.fcs.tif | rpt_231-25-2.fcs.tif | rpt_231-25-3.fcs.tif | rpt_231-control-1.fcs.tif

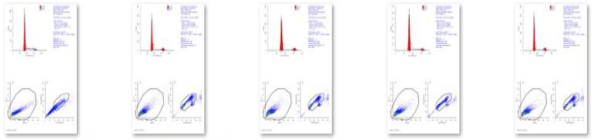

rpt_231-control-2.fcs.tif | rpt_231-control-3.fcs.tif | rpt_231-dmso-1.fcs.tif | rpt_231-dmso-2.fcs.tif | rpt_231-dmso-3.fcs.tif

MDA-MB-468

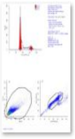
rpt_468-15-1.fcs.tif

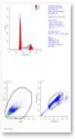
rpt_468-15-2.fcs.tif

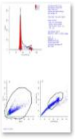
rpt_468-15-3.fcs.tif

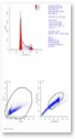
rpt_468-20-1.fcs.tif

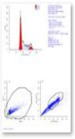
rpt_468-20-2.fcs.tif

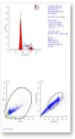
rpt_468-20-3.fcs.tif

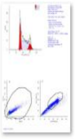
rpt_468-25-1.fcs.tif

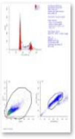
rpt_468-25-2.fcs.tif

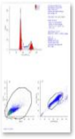
rpt_468-25-3.fcs.tif

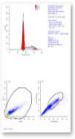
rpt_468-control-1.fcs.tif

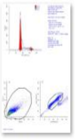
rpt_468-control-2.fcs.tif

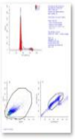
rpt_468-control-3.fcs.tif

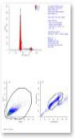
rpt_468-dmso-1.fcs.tif

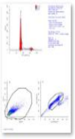
rpt_468-dmso-2.fcs.tif

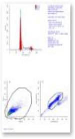
rpt_468-dmso-3.fcs.tif

7. qRT-PCR

1.

1	CT					target gene-GAPDH				Relative expression value		
	GAPDH	plk1	ccnb1	p53		plk1	ccnb1	p53		plk1	ccnb1	p53
blank	21.20	25.63	26.02	27.02	blank	4.43	4.82	5.82	blank	101.892	102.747	102.747
	21.20	25.73	26.06	27.06		4.53	4.86	5.86		95.465	100	100
	21.10	25.57	26.08	27.02		4.46	4.97	5.91		100	92.5353	96.4649
dmso	21.31	25.67	26.05	27.05	dmso	4.36	4.74	5.74	dmso	107.232	108.846	108.846
	21.27	25.88	26.19	27.19		4.60	4.92	5.92		90.5674	95.9938	95.9938
	21.21	25.60	26.01	27.01		4.38	4.79	5.79		105.565	104.767	104.767
15	20.78	25.67	26.16	24.96	15	4.89	5.38	4.18	15	74.2221	69.6902	320.212
	20.80	25.62	26.10	25.00		4.82	5.30	4.20		78.0609	73.9167	316.888
	20.81	25.61	26.33	24.93		4.80	5.52	4.12		79.0113	63.329	334.253
20	21.35	28.22	27.66	24.36	20	6.87	6.31	3.01	20	18.7765	36.6009	720.977
	21.32	28.06	27.72	24.42		6.73	6.40	3.10		20.6836	34.434	678.291
	21.26	28.11	27.66	24.26		6.85	6.41	3.01		19.0561	34.2132	722.314
25	20.34	27.53	27.43	23.18	25	7.20	7.10	2.84	25	15.019	21.2401	811.04
	20.40	27.58	27.58	23.07		7.18	7.18	2.66		15.1552	19.9974	916.877
	20.22	27.36	27.36	23.01		7.14	7.14	2.80		15.5993	20.5834	835.003

2.

2	CT					target gene-GAPDH				Relative expression value		
	GAPDH	plk1	ccnb1	p53		plk1	ccnb1	p53		plk1	ccnb1	p53
blank	21.42	24.07	23.77	23.23	blank	2.65	2.35	1.81	blank	113.485	100.976	109.255
	21.20	24.14	23.56	23.20		2.94	2.36	2.00		92.8378	100	95.837
	21.24	24.07	23.61	23.19		2.83	2.36	1.94		100	99.7525	100
dmso	21.49	24.29	23.81	23.56	dmso	2.81	2.32	2.07	dmso	101.703	103.014	91.1707
	21.47	24.29	23.87	23.45		2.82	2.40	1.99		100.584	97.121	96.7096
	21.42	24.23	23.69	23.29		2.81	2.27	1.86		101.535	106.417	105.424
15	22.07	25.58	25.04	22.45	15	3.52	2.98	0.39	15	62.0875	65.1413	293.645
	22.06	25.40	25.06	22.34		3.34	3.00	0.28		70.4451	64.1056	316.969
	21.88	25.40	24.88	22.06		3.53	3.00	0.19		61.6614	63.9764	337.486
20	22.37	27.45	26.25	21.54	20	5.08	3.87	-0.83	20	21.0217	35.0503	682.947
	22.35	27.47	26.26	21.47		5.12	3.91	-0.88		20.4378	34.0691	706.389
	22.25	27.59	26.34	21.37		5.34	4.09	-0.87		17.5177	30.0905	702.373
25	21.87	27.50	26.33	20.74	25	5.63	4.46	-1.13	25	14.3427	23.3276	840.716
	21.97	27.72	26.60	20.90		5.75	4.63	-1.07		13.1765	20.7298	806.647
	21.91	27.63	26.63	20.88		5.72	4.73	-1.02		13.4864	19.3513	780.659

3.

3	CT					target gene-GAPDH				Relative expression value		
	GAPDH	plk1	ccnb1	p53		plk1	ccnb1	p53		plk1	ccnb1	p53
blank	21.33	23.02	24.43	28.01	blank	1.69	3.10	6.68	blank	100	100	97.26549
	21.4	23.12	24.3	28.00		1.72	2.90	6.60		97.94203	114.8698	102.8114
	21.25	22.80	24.45	27.89		1.55	3.20	6.64		110.1905	93.3033	100
dmso	21.67	23.45	24.5	27.99	dmso	1.78	2.83	6.32	dmso	93.95227	120.5808	124.8331
	21.8	23.50	24.86	28.32		1.70	3.06	6.52		99.30925	102.8114	108.6735
	21.43	23.12	24.55	28.24		1.69	3.12	6.81		100	98.62327	88.88427
15	21.34	23.58	25.04	26.37	15	2.24	3.70	5.03	15	68.30201	65.9754	305.2518
	21.56	23.60	25.24	26.70		2.04	3.68	5.14		78.45841	66.89638	282.8427
	21.5	23.61	25.22	26.41		2.11	3.72	4.91		74.74246	65.06709	331.7278
20	21.65	25.67	26.49	25.63	20	4.02	4.84	3.98	20	19.88841	29.93697	632.033
	21.48	25.47	26.35	25.57		3.99	4.87	4.09		20.30631	29.32087	585.6343
	21.62	25.66	26.35	25.31		4.04	4.73	3.69		19.6146	32.30882	772.7491
25	21.71	26.08	27.19	25.30	25	4.37	5.48	3.59	25	15.60413	19.21094	828.2119
	21.74	26.21	27.02	25.36		4.47	5.28	3.62		14.55917	22.06757	811.1676
	21.67	26.14	27.16	25.14		4.47	5.49	3.47		14.55917	19.07824	900.0468

8. Western blot

1.

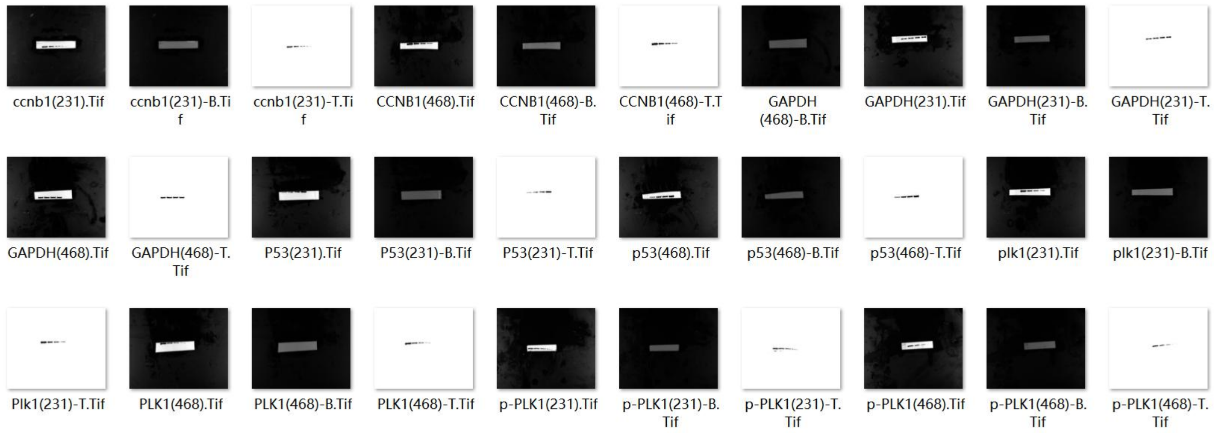

2.

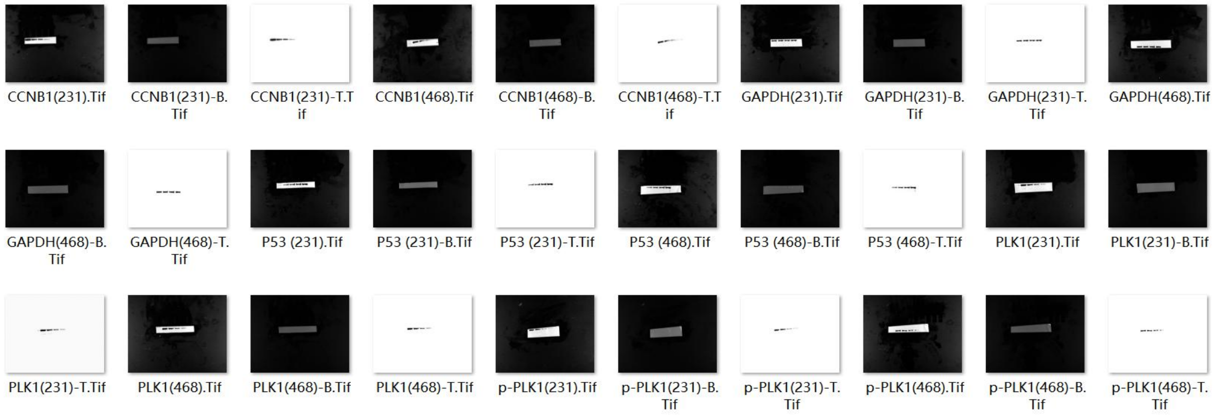

3.

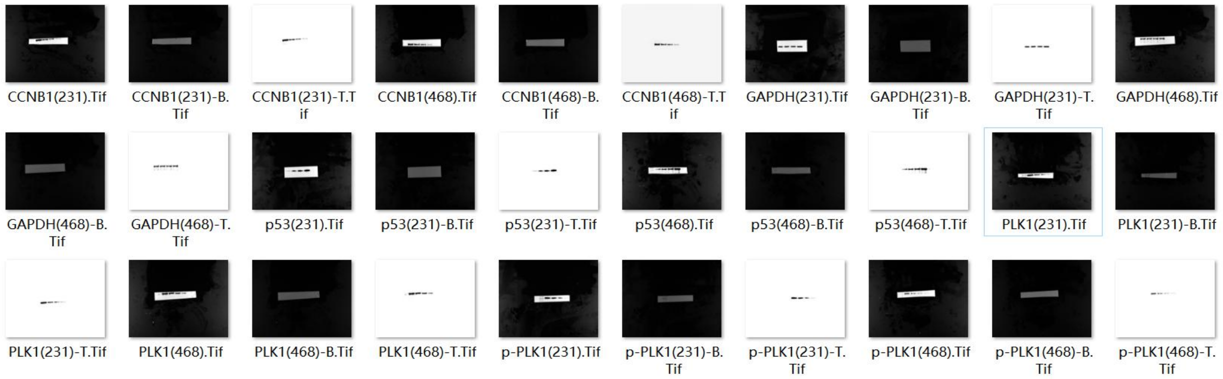

Supplement: Supplementary file 1 [file DataSheet1.PDF]
